# The Role of Surface Complexes in Ketene Formation from Fatty Acids via Pyrolysis over Silica: from Platform Molecules to Waste Biomass

**DOI:** 10.1021/jacs.3c06966

**Published:** 2023-12-04

**Authors:** Liana R. Azizova, Tetiana V. Kulik, Borys B. Palianytsia, Mykola M. Ilchenko, German M. Telbiz, Alina M. Balu, Sergiy Tarnavskiy, Rafael Luque, Alberto Roldan, Mykola T. Kartel

**Affiliations:** † School of Dentistry, Cardiff University, Heath Park, Cardiff CF14 4XY, U.K.; ‡ Chuiko Institute of Surface Chemistry, National Academy of Science of Ukraine, Kyiv 03164, Ukraine; § Cardiff Catalysis Institute, School of Chemistry, Cardiff University, Main Building, Park Place, Cardiff CF10 3AT, U.K.; ∥ Departamento de Química Orgánica, Universidad de Córdoba, Campus de Rabanales, Edificio Marie Curie (C-3), Ctra Nnal IV-A, Km 396, Cordoba E14014, Spain; ⊥ Institute of Molecular Biology and Genetics, National Academy of Science of Ukraine, 150 Zabolotnogo Str., Kyiv 03680, Ukraine; # National Academy of Science of Ukraine, L. V. Pisarzhevsky Institute of Physical Chemistry, Nauky Av. 31, Kyiv 03039, Ukraine; ∇ Universitá degli studi Mediterranea di Reggio Calabria (UNIRC), DICEAM, Via Zehender (giá via Graziella), Loc. Feo di Vito, I89122 Reggio Calabria, Italy; ○ Universidad ECOTEC, Km. 13.5 Samborondón, Samborondón EC092302, Ecuador

## Abstract

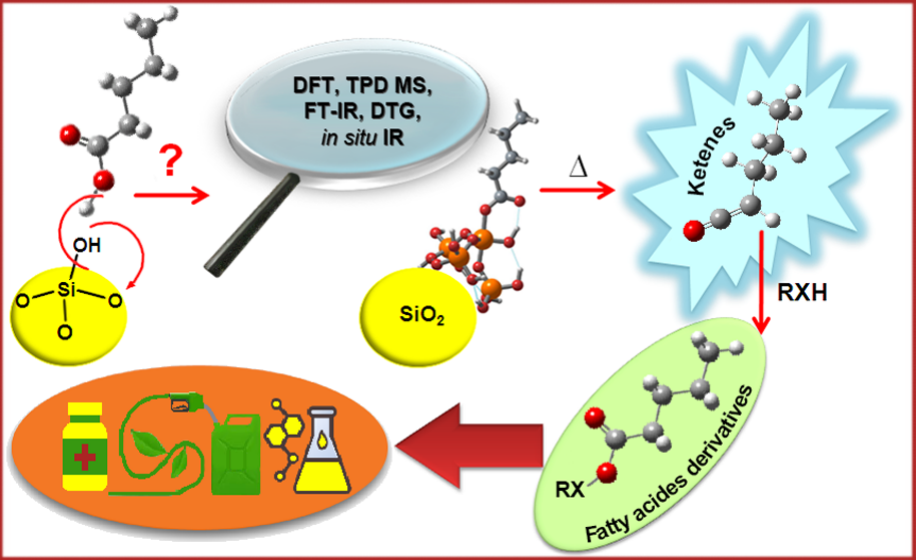

Fatty acids (FA) are the main constituents of lipids and oil crop waste, considered to be a promising 2G biomass that can be converted into ketenes via catalytic pyrolysis. Ketenes are appraised as promising synthons for the pharmaceutical, polymer, and chemical industries. Progress in the thermal conversion of short- and long-chain fatty acids into ketenes requires a deep understanding of their interaction mechanisms with the nanoscale oxide catalysts. In this work, the interactions of fatty acids with silica are investigated using a wide range of experimental and computational techniques (TPD MS, DFT, FTIR, *in situ* IR, equilibrium adsorption, and thermogravimetry). The adsorption isotherms of linear and branched fatty acids C1–C6 on the silica surface from aqueous solution have been obtained. The relative quantities of different types of surface complexes, as well as kinetic parameters of their decomposition, were calculated. The formation of surface complexes with a coordination bond between the carbonyl oxygens and silicon atoms in the surface-active center, which becomes pentacoordinate, was confirmed by DFT calculations, in good agreement with the IR feature at ∼1680 cm ^1^. Interestingly, ketenes release relate to these complexes’ decomposition as confirmed by the thermal evolution of the absorption band (1680 cm^–1^) synchronously with the TPD peak of the ketene molecular ion. The established regularities of the ketenezation are also observed for the silica-induced pyrolysis of glyceryl trimyristate and real waste, rapeseed meals.

## 1 Introduction

Interest in the industrial application of vegetable oils and fats for developing renewable energy sources, biofuels, and oleochemicals has rapidly increased in recent years.^1^ The hydrolysis of triglycerides into glycerine and mixed fatty acids is a major process to convert biomass into biobased chemicals and fuels.^2^ Pyrolysis and thermochemical conversion of vegetable oils and fats into oleochemicals and fuels is inexpensive and requires mild temperatures.^3,4^

Fatty acids (FA) are main constituents of vegetable oils and fats.^2,5^ Short-chain acids (ethanoic, butanoic, pentanoic, 4-oxopentanoic acid (levulinic), etc.) and various furan derivatives with a carboxyl group (furandicarboxylic acid, etc.) can be obtained in large volumes through biochemical and chemical catalysis of lignocellulosic biomass.^6,7^ The short- and long-chain fatty acids and some other acids are used as platform molecules in biomass conversion technologies and as building blocks in producing pharmaceuticals, polymers, and fine chemicals.^8,9^

The catalytic pyrolysis of carboxylic acids is an environmentally benign, one-step process that avoids toxic reagents and solvents and does not generate harmful byproducts.^6,10^ The most common catalysts are inorganic oxides based on Al, Si, Ti, Zr, Ce, Zn, Mg, Fe, Ni, Co, etc.^11,12^ These catalysts are employed to obtain alkanes, aldehydes, ketones, and ketenes.^12−15^ Among these inorganic supports, silica and its derivatives with controlled particle size, morphology, and surface area provide good mechanical properties, thermal stability, accessible functionalization properties, and resistance against attacks by organic solvents, making them popular catalysts for pyrolysis.^16^

Ketenes are widely used in industrial chemistry.^17^ The catalytic production of ketenes by pyrolysis of carboxylic acids proceeds at lower temperatures and is less harmful to the environment than traditional synthesis methods.^18,19^ The traditional methods of ketene production, such as thermal pyrolysis of carboxylic acids and ketones, dehalogenation of α-halo acyl halides, and dehydrohalogenation of acyl halides with tertiary amines in a solvent, are multistep processes with the utilization of hazardous reagents and solvents producing chlorinated byproducts.^20^ Therefore, ketene production by the catalytic pyrolysis of carboxylic acids over silica is an environmentally benign alternative.^21^ Thus, investigation of the catalytic pyrolysis mechanism of carboxylic acids over silica is essential for further developing oleochemical production methods.

Ketenes attract significant interest as synthons in organic synthesis due to their high reactivity and nucleophilicity.^17,22^ Importantly, ketenes are considered privileged synthons in the syntheses of three-, four-, five-, and six-membered heterocycles.^23^ They can be used to synthesize any organic acid derivatives and as acylating agents,^24^ for example, in the catalytic acylation of anisole by acetic anhydride to yield *p*-methoxyacetophenone.^25,26^ Ketenes are also used in pharmaceutical applications such as preparing β-lactam antibiotics.^27^

In addition, establishing the critical role of ketenes as an essential member of the highly reactive first-generation reaction intermediates such as carbocations, carbanions, radicals, and carbenes has been the target in a number of studies mainly during the last five years.^28−32^ Their crucial role in processes of zeolite catalysis was identified as (1) syngas to light olefins over oxide–zeolite-based composites;^31^ (2) methanol conversion to hydrocarbons;^29^ (3) catalytic acylation of anisole by acetic anhydride to yield *p*-methoxyacetophenone;^25^ (4) ketene transformation to gasoline;^30^ (5) ketonization of aliphatic acids;^32^ and (6) acid-catalyzed carbonylation over zeolites.^33^ Chen and coauthors showed that ketenes are intermediates in the acid-catalyzed carbonylation over zeolites by *ab initio* molecular dynamics simulations and the confinement effect enforced by the pores of zeolites significantly influences this reaction (via formation and stability of ketenes^33^). The role of ketenes was revealed as a critical intermediate and product in the pyrolysis processes of biomass and its model compounds, i.e., cinnamic acids.^34,35^

The usage of a variety of quantum-mechanical methods, including density functional theory (DFT) to model silica surfaces was discussed by Rimola et al.^36^ The established patterns of catalytic pyrolysis can help to select conditions of the process, inorganic oxides, to obtain the desirable product in quantitative yields. In addition, establishing binding mechanisms with the surface is practical in method development solving urgent problems in biology and medicine. Indeed, the carboxyl group is the primary building block of practically all essential biomolecules (amino acids, peptides, proteins, lipids, etc.). Although many studies investigate carboxylic acid decomposition on the silica surface, adsorption mechanisms, and the surface intermediates, these brought to date no clear conclusion. The influence of hydrophilic and hydrophobic interactions and hydrocarbon chain length on adsorption mechanisms could not be established as well as mechanisms of ketene formation on the silica surface and the influence of the structure of acids on them. Hence, an endeavor has been undertaken to investigate the kinetics and mechanisms governing chemical reactions involving carboxylic acids on the surface layer of silica materials. The objective is to ascertain the adsorption structures of the complexes formed and understand the underlying mechanisms in the interaction between substances containing a carboxyl group and active sites present on the silica surface.

## 2 Experimental Section

### 2.1 Reagents

Fumed silica A-300 (pilot plant at the Chuiko Institute of Surface Chemistry, Kalush, Ukraine; specific surface area of 270 m^2^·g^–1^) was used in this work. Fumed silica was previously heated on air for 2 h at 400 °C for removal of adsorbed organic substances. The aliphatic carboxylic acids were obtained from Merck, Germany (methanoic acid (formic), ethanoic acid (acetic), propanoic acid (propionic), butanoic acid (butyric), 2-methylpropanoic acid (isobutyric), pentanoic acid (valeric), hexanoic (caproic), octadecanoic acid (stearic), 99.5%), and Fluka, Germany (2,2-dimethylpropanoic acid (pivalic acid), 99.5%). Glyceryl trimyristate was obtained from Merck, Germany (≥99%). Rapeseed meal used in this work was obtained from the Kyiv region of Ukraine.

### 2.2 Loading FAs on the Silica Surface

#### 2.2.1 Equilibrium Adsorption Procedure

A 100 mg portion of silica was immersed in an aqueous solution of suitable acid of the appropriate concentration (0.01–0.1 mol·L^–1^) at pH 3.2 ± 0.3 and stirred at room temperature for 24 h to achieve equilibrium. The suspension was decanted and centrifuged for 20 min at 10.000 rpm until the supernatant is clear. Then, samples were dried in air at room temperature. The samples were then aged at room temperature for 24 h, dried, and stored in a desiccator. An adsorption value was determined by difference in the aqueous phase concentration of the organic acids before and after adsorption. The concentration of acids in solutions has been determined by titration with NaOH solution using phenolphthalein as the indicator before and after adsorption.

The adsorption capacity of silica toward FAs in mg·g^–1^ was calculated as
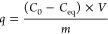
where *C*_0_ and *C*_eq_ correspond to the initial and equilibrium concentrations of FA, respectively, (mg·L^–1^); *V* corresponds to the volume of the acid aqueous solution (mL); and *m* corresponds to the mass of sorbent (g).

#### 2.2.2 Gas-Phase Adsorption

Adsorption of acid molecules was provided by expanded vapors of pentanoic acid on the preliminary outgassed (450 °C, 133 Pa, 2h) thin self-supporting wafer SiO_2_ sample. Prior to adsorption, pentanoic acid was degassed by online freeze–pump–thaw cycles performed at liquid nitrogen temperature.

#### 2.2.3 Modification by Impregnation

Additionally, other samples with concentrations of 0.1, 0.3, 0.4, and 0.6 mmol·g^–1^ of the corresponding acid on the silica were obtained by impregnation. A 25 mL of aqueous solution of acid was added to 1 g of fumed silica in Petri dish. A sample of hexanoic acid due to its low solubility in water was obtained from aqueous ethanol solution (50% alcohol by volume) of silica. The silica-supported acid samples for thermogravimetric analysis were obtained by soaking silica in CCl_4_ solutions of the corresponding acid. The stearic acid and glyceryl trimyristate samples (0.3 mmol·g^–1^) on the silica surface were obtained by soaking silica in their ethanol solutions. The components were mixed and left on air at ∼20 °C until the solvent was fully evaporated (∼24 h). Mechanical mixtures of rapeseed meal with silica were obtained by grinding in an agate mortar. In experiment, an air-dried sample was under investigation. The samples were then aged at room temperature for 24 h, dried, and stored in a desiccator.

### 2.3 Fourier Transform Infrared Spectroscopy (FTIR)

FTIR-spectra were recorded at room temperature in the range between 400 and 4000 cm^–1^ on a Thermo Nicolet NEXUS FT-IR spectrophotometer. The spectra were obtained with a theoretical resolution of 4 cm^–1^ with 100 scans and a scan speed of 0.5 cm·s^–1^. FTIR spectra were obtained for all samples (adsorbed and impregnated). Both the self-supporting discs (pellets) prepared by pressing, using 8 MPa pressure, weighing about 20 mg, and the powder weighing about 180 mg were used in the IR spectrometric measurement. The weights of silica samples were 20 (for pellets) and 180 mg (for powder).

Pellets of silica-supported acids with a density of 2–3 mg·cm^–2^ for FTIR investigations with heating and evacuation steps were placed in a specially designed cell attached to the vacuum system. The cell was designed in way allowed carry out heating and further evacuation without change arrangement of sample in the cell. The sample was evacuated at room temperature and a pressure of 10^–4^ mmHg for 3 h before measurements. Spectra were taken after evacuation at room temperature and 60 and 100°C on a Specord-75 IR spectrometer (“Carl Zeiss”, Germany).

### 2.4 *In Situ* IR Spectroscopy

A heatable and stationary mounted quartz glass IR cell equipped with cooled KBr windows was used for the examination of adsorbed species produced as a result of surface reactions of valeric acid molecules on the active centers of SiO_2_ following a temperature treatment at desired temperatures. Specord IR-75 (“Carl Zeiss”, Germany) was the employed spectrometer. The spectra were recorded after outgassing samples at the desired temperature (kept for 5 min) and cooling to RT. For these studies, samples of pentanoic acid obtained by adsorption from the gas phase and by the impregnation method (0.3 mmol·g^–1^) were used.

### 2.5 Method of Temperature-Programmed Desorption Mass Spectrometry

The method of conducting the TPD MS experiment was described in detail in a number of our previous publications,^37−39^ as well as obtaining kinetic parameters from the TPD MS data.^38,39^

### 2.6 Thermogravimetric Analysis

Thermogravimetric analyses of samples were run on a thermogravimetric analyzer TGA-6 (PerkinElmer, USA) in an oxygen atmosphere (flow rate = 20 cm^3^·min^–1^). In a typical run, 20 mg of the sample was heated from 30 to 700°C at a heating rate of 10°C·min^–1^.

### 2.7 Quantum Chemical Methods

The density functional theory method (DFT) was used to identify the nature of the absorption bands in the IR spectra. DFT calculations were carried out using the B3LYP, wB97XD, and M062X functionals and the 6-311++G(d,p) basis set. The last two functionals include the dispersion correction of forces, which is especially important in the study of intermolecular interactions. All calculations were performed using the GAUSSIAN 09 software (version D.01).^40^ Active centers of the silica surface on which the adsorption and further thermal transformations of valeric acid probably occur were selected within^41^ the framework of the cluster approximation model. In this approximation, the broken chemical bonds at the silica active centers, directed into the volume of the solid, were closed by hydrogen atoms.

In addition, the silica surface itself was modeled using two different approaches. The first one is based on the “soft” surface model, in which the SiO_4_ fragments in the active center can move relatively easily relative to each other. In this case, the structural rigidity (the possibility of rearrangement) is determined mainly by the energies of hydrogen bonds and dispersive interactions. The second approach is the so-called “hard” surface model. The possibility of the active center reorganization is determined primarily by the deformation of Si–O–Si and O–Si–O valence angles and the change in the torsion angles between its fragments.

## 3 Results and Discussion

Establishing the interaction between the silica surface and compounds containing a carboxyl group is of significant fundamental and applied importance. Ketenization reaction kinetics for a series of aliphatic acids on a silica surface was previously investigated using the linear free energy relationships (LFER) approach.^19^ Kinetic parameters and reaction constants, ρ_0_, were calculated for the reaction of ketene formation. Based on the data obtained, an intramolecular mechanism of ketenization was proposed from an adsorbed carboxylate, which proceeds through a four-membered low-polarity transition state. However, the question regarding the adsorbed carboxylate remains open mainly because there is no sufficient spectroscopic confirmation of such species, e.g., the corresponding IR absorption bands. Despite surface siloxane bonds being nonactive,^19,38^ silanol groups lead to carboxylates upon interaction with the acidic substrate leading to an absorption band ν_C=O_ around 1680 cm^–1^ for adsorbed valeric acid.^15,21,42,43^ An alternative adsorption mechanism was proposed resulting from a nucleophilic addition to siloxane bridges.^19,35^ Zhuravlev^44^ considers the formation of reactive siloxane bridges resulting from surface dehydration and dehydroxylation processes as confirmed in several simulation works.^45^ These studies provide data on the participation of siloxane bridges in nucleophilic addition reactions, particularly with ammonia and some primary, secondary, and tertiary amines.^46^ The data of TPD MS studies^19^ indicate a more complex nature of the interaction of aliphatic acids with the silica surface. In particular, three stages of desorption and destruction of carboxylic acids from the surface indicate at least three surface forms of adsorption. Obviously, the silica surface cannot be described only by the ideal cristobalite structure with uniformly distributed silanols; i.e., the surface may present areas of unevenly distributed silanol groups and hydrophobic siloxane. The following IR spectroscopy adsorption data and computational simulations correlate the stages during the pyrolytic process to specific adsorption modes of carbolylic structures on silica surfaces, including forming donor–acceptor intermediate complexes between the carboxylic substrates and a penta-coordinated silicon atom.

### 3.1 Adsorption Properties

**Figure 1:**
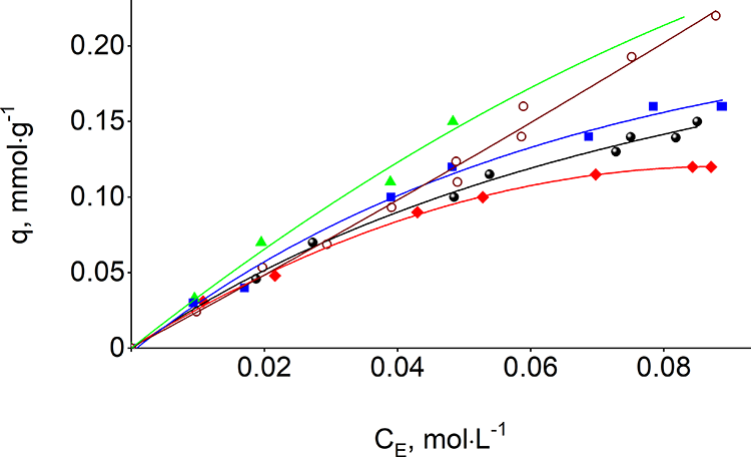
Adsorption isotherms of methanoic acid (circle solid), ethanoic acid (red tilted square solid), propanoic acid (green triangle up solid), butanoic acid (blue box solid), pentanoic acid (circle open) on the silica surface from aqueous solution.

**Figure 2:**
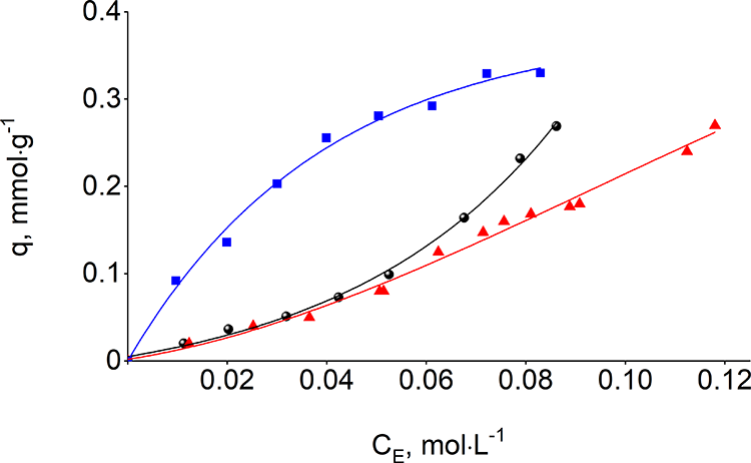
Adsorption isotherms of 2-methylpropanoic acid (circle solid), 2.2-dimethylpropanoic acid (red triangle up solid), and hexanoic acid (blue box solid) on the silica surface from aqueous solution.

The carboxylic substrates’ adsorption data (Figures 1 and 2 and Table 1) is well described by the Langmuir isotherm model (*R*^2^ = 0.9534–0.9897), leading to an L-shape for adsorption isotherms of linear acid isomers and an S-shape for branched-chain isomers. Values of equilibrium adsorption capacity (*q*_max_), adsorption equilibrium constant (*K*), and Gibbs free energies (Δ*G*) were obtained from linear regression analysis of known Ce and *q* values^47,48^ and are listed in Table 1. The equilibrium adsorption capacities, proportional to the concentration of free silanol groups on the fumed silica surface (0.6 mmol·g^–1^), expand between 0.2 and 0.5 mmol·g^–1^. The isotherm-derived Gibbs free energies for propanoic, butanoic, and pentanoic acids have very close values, indicating a low affinity between carboxylic acids to the silica surface.

**Table 1:** Physico-Chemical Parameters of Adsorption of Carboxylic Acids on the Silica Surface (Obtained by Linear Regression Analysis for Langmuir Adsorption Isotherm)

acid	*q*_max_/ mmol·g^–1^	K/L·mol^–1^	Δ*G*/ kJ·mol^–1^	*R*^2^
methanoic (formic)	0.36	8.02	–4.73	0.9860
ethanoic (acetic)	0.21	15.7	–6.2	0.9897
propanoic (propionic)	0.42	9.2	–5.0	0.9891
butanoic (butyric)	0.28	6.6	–4.3	0.9846
pentanoic (valeric)	0.33	6.9	–4.4	0.9534
2,2-dimethylpropanoic (pivalic)	0.25	1.61	–1.1	0.9879
hexanoic (caproic)	0.50	22.3	–7.04	0.9807

### 3.2 FT-IR

#### 3.2.1 FT-IR Investigation of Carboxylic Acids Adsorbed on the Silica Surface from the Aqueous Phase

**Scheme 1:**
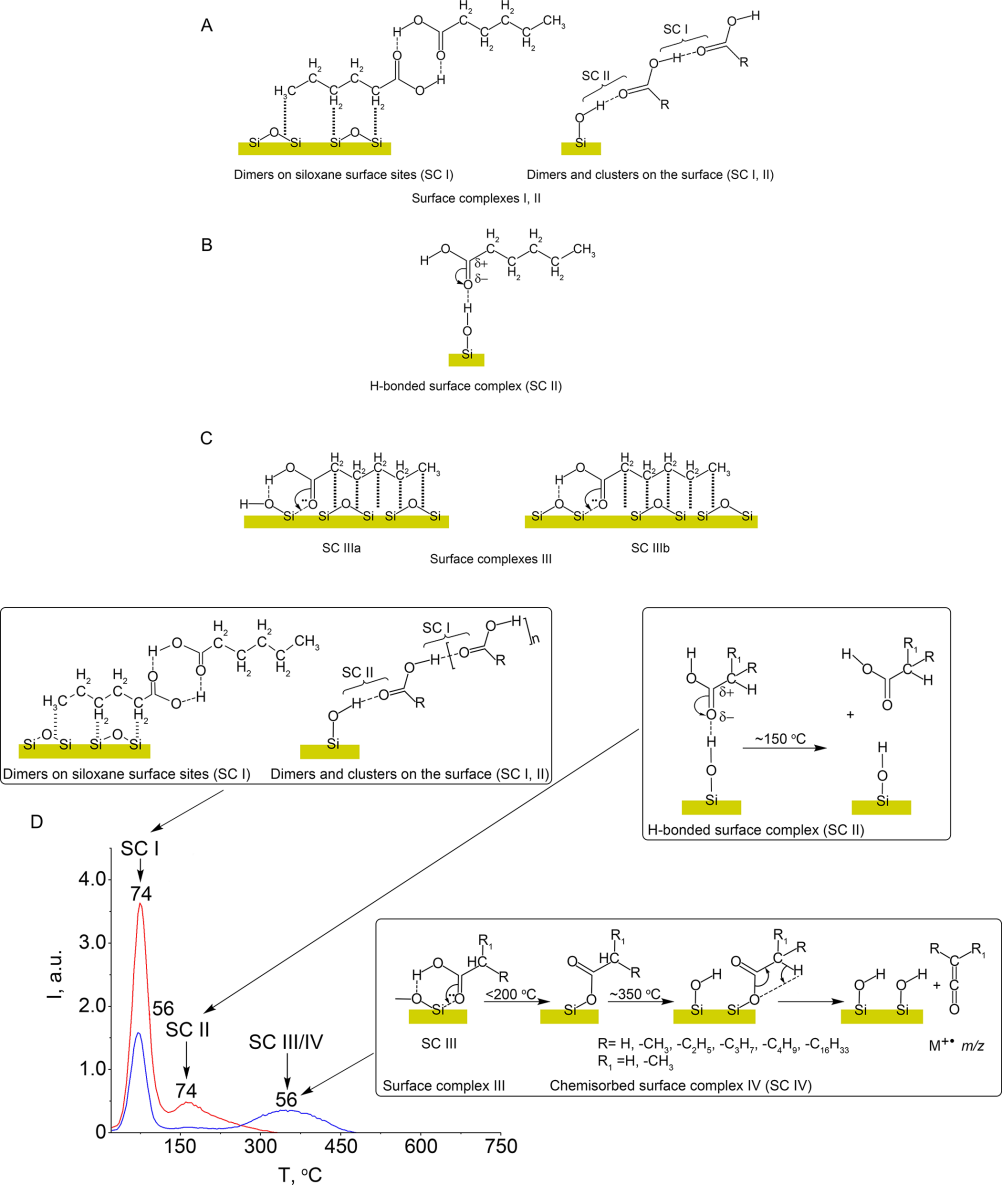
Surface Complexes of Carboxylic Acids on the Silica Surface (A) dimers and clusters, physically adsorbed complexes of acid, SC I; (B) hydrogen-bonded complexes SC II; (C) donor–acceptor coordination complexes SC IIIa and SC IIIb. (D) TPD-peaks related to the thermal decomposition of the surface complexes SC I, SC II, SC IIIa, and chemosorbed complex SC IV, which decomposes with ketene formation.

Upon silica exposure to the carboxylic substrates, FT-IR spectra were investigated in order to determine the adsorption modes and the complexes’ structure. The observed vibration of the carboxylic ν_C=O_ stretching in saturated fatty acids is more intense than in ketones, appearing in the 1725–1705 cm^–1^ range (Table 2). These are comparatively lower to isolated saturated aliphatic acids in inert solvents (1760 cm^–1^) due to the hydrogen bonds formed between carboxylic acid dimers.^49^ No bands could be attributed to unassociated carbonyl groups, typical monomeric acids, on samples obtained by the impregnation method (0.2–0.5 mmol·g^–1^). The absence of any vibration bands at 1760 cm^–1^ suggests that carboxylic acid is not free but rather associated. Indeed, the intensity of stretching vibrations of isolated silanols at 3745 cm^–1^ decreased for all investigated acids, confirming the formation of hydrogen-bonded adsorbed complexes SC II (Scheme 1). The stretching band at 3745 cm^–1^ did not completely disappear (Figure 2), meaning that not all isolated silanols (∼20%) participate in the formation of adsorption complexes with carboxylic acids. The dependence of surface coverage versus acid surface concentration was calculated from the band’s intensity for hexanoic and propanoic acids. Ca. 20% of silanols formed the surface complex even at high surface coverage.

**Table 2:** Absorption Frequencies of the Carbonyl Group ν_(C=O)_ for Silica-supported Samples of Hexanoic Acid and Corresponding Shift Values Δν_(C=O)_ Relative to ν_(C=O)_ of Monomer and Dimer^a^

sample of acid	acid solution in CCl_4_ (monomer)^49^	acid solution in CCl_4_ (dimer)^49^	acid adsorbed on the silica surface from aqueous solution			acid immobilized on the silica surface by impregnation (0.6 mmol·g^–1^)		
	ν_C=O_, cm^–1^	ν_C=O_, cm^–1^	ν_C=O_, cm^–1^	Δν_1_, cm^–1^	Δν_2_, cm^–1^	ν_C=O_, cm^–1^	Δν_1_, cm^–1^	Δν_2_, cm^–1^
hexanoic (caproic) 0.503 mmol·g^–1^	1757	1711	1686	+25	+71	1685	+26	+72
			1703	+8	+54	1705	+6	+52
						1716	–5	+41
						1732	–21	+25

a Δν_1_ = ν_dimer_ – ν_ads._ – absorption frequencies, ν_C=O_ shift of adsorption complex relative to the dimer. Δν_2_ = ν_monomer_ – ν_ads._ – absorption frequencies, ν_C=O_ shift of adsorption complex relative to monomer.

A ν_C=O_ frequency blue-shift (higher frequencies) relative to dimer stretching of Δν_1_ = 21 cm^–1^ (Δν_1_ = ν_dimer_ – ν_ads.ac._) is observed for hexanoic acid, opposite to the red-shift of Δν_1_ = 34 cm^–1^ for propanoic acid (Table 2).

**Figure 3:**
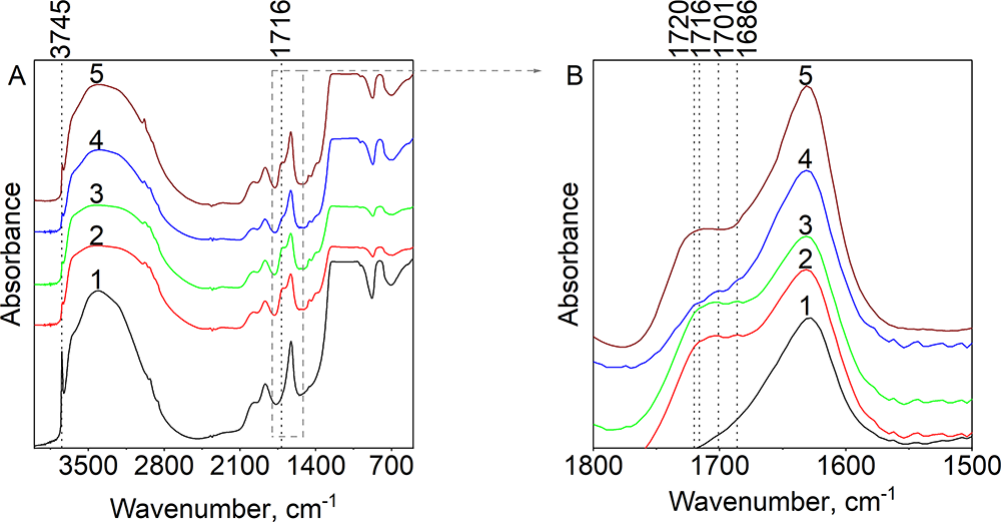
IR-spectra in the range of (A) 4000–400 and (B) 1800–1500 cm^–1^ of silica (line 1) and silica-supported acids (adsorbed): hexanoic (0.503 mmol·g^–1^; line 2), pentanoic (0.327 mmol·g^–1^; line 3), butanoic (0.282 mmol·g^–1^; line 4), and propanoic (0.42 mmol·g^–1^; line 5).

**Figure 4:**
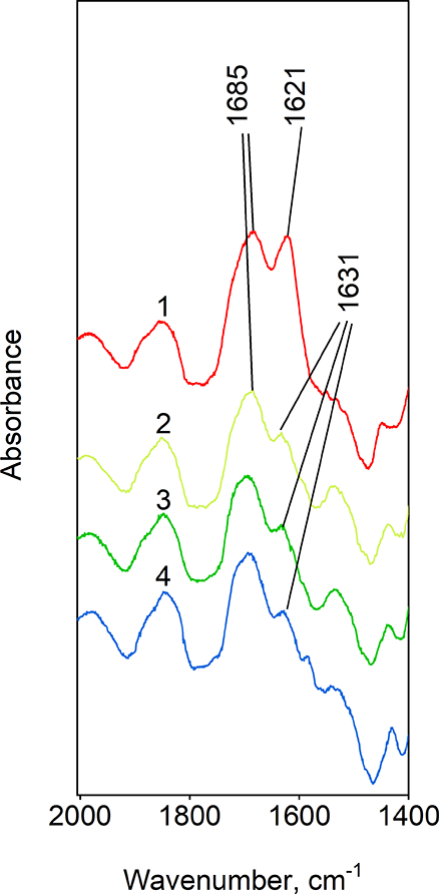
*In situ* IR spectroscopy. IR spectra of pentanoic acid on the surface of SiO_2_ (0.3 mmol·g^–1^): (1) at room temperature; (2) after evacuation; (3) after heating to 60 °C; and (4) after heating to 100 °C.

A shift to higher frequency indicates the formation of hydrogen-bonded adsorbates SC II (Scheme 1) due to the weaker hydrogen bond between silanol groups (Scheme 1); red-shifts point toward the suggested formation (Scheme 1; SC IIIa, SC IIIb). Indeed, considering the ν_(C=O)_ shift (Δν_2_ = ν_monom._ – ν_ads.ac._) relative to monomeric carboxylic acid at 1757 cm^–1^, it is possible to assign, for instance, the hexanoic acid ν_C=O_ bands to Δν_2C=O_ = 71 cm^–1^ – adsorption complex SC IIIa (the Scheme 1); Δν_2C=O_ = 54 cm^–1^ – adsorption complex SC IIIb (Scheme 1); Δν_2C=O_ = 41 cm^–1^ – dimer of acid SC I (Scheme 1); Δν_2C=O_ = 25 cm^–1^ – hydrogen-bonded complex SC II (Scheme 1). Similarly, the ν_C=O_ stretching band for the adsorbed butanoic acid sample is suggested to deconvolute in three interactions, whereas they are four for adsorbed propanoic and hexanoic acid samples (Figures 3 and 4 and Table 2). Such IR band deconvolution of silica-adsorbed species is explained by various adsorption complexes, as previously reported for carboxylic acids, ketones, and esters.^47,50^ The most characteristic vibrational bands corresponding to surface complexes SC IIIa,b are at 1697–1705 cm^–1^, corresponding to low and high coverages (Figures S1 and S2 (Supporting Information)). While similar shifts of the bands of carbonyl group ν_C=O_ were observed for all carboxylic acids, Table 2 presents data specifically for hexanoic acid in order to illustrate the aforementioned discussion.

#### 3.2.2 *In situ* IR Study of Valeric Acid Conversion over Silica obtained from Gas Phase and by Impregnation from Aqueous Solution

The IR of pentanoic acid on silica shows a relevant band at 1685 cm^–1^ corresponding to surface complexes SC IIIa,b and can be observed under various conditions: room temperature and atmospheric pressure and after evacuation in vacuum at 20, 60, and 100 °C (Figure 4). While the intensity of such band remains upon evacuation in a vacuum, the vibration related to physically adsorbed water at 1621–1631 cm^–1^ significantly decreases (although still observable under vacuum at 100 °C due to the hydroxyl groups on the surface). The vibrational band at 1621–1631 cm^–1^ in the FTIR spectrum is attributed to the combined contributions of strongly bound water within voids and physically adsorbed water on the silica’s surface. While the band’s intensity remains upon evacuation in a vacuum due to the presence of strongly bound water within voids, the hydroxyl groups on the surface allow for the continued observation of the band related to physically adsorbed water, even at elevated temperatures under vacuum conditions.

**Figure 5:**
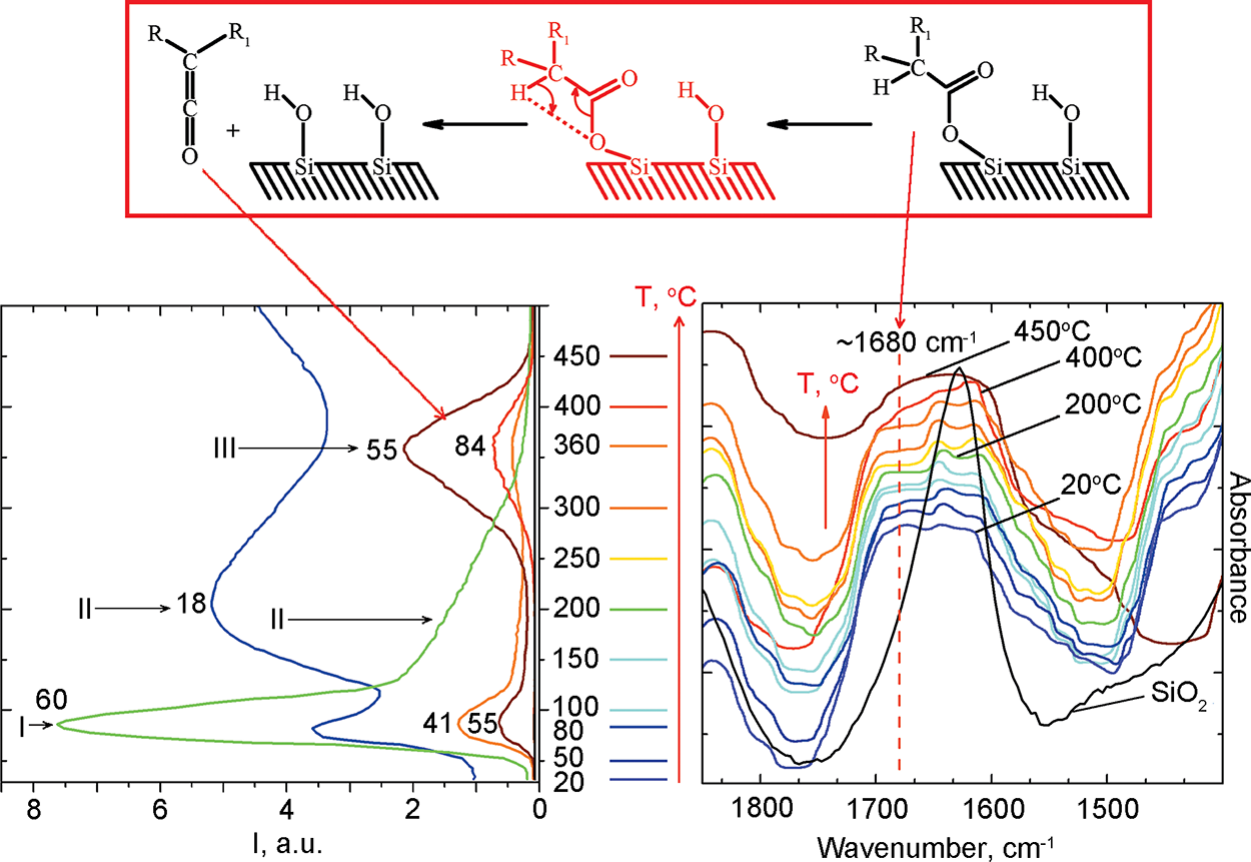
*In situ* IR spectroscopy and TPD-curve for molecular and fragment ions of propylketene (*m*/*z* 84, 55, 41), fragment ions of valeric acid (*m*/*z* 60, 55, 41), and molecular ion of water (*m*/*z* 18) obtained during pyrolysis of valeric acid over nanosilica.

Monodentately bound complexes of fatty acids on the surface of silica are challenging to identify employing IR spectroscopy since their ν_C=O_ absorption region at ∼1680 cm^–1^ is overlapped with the ν_C=O_ band (∼1715–1730 cm^–1^) and the water δ_(H–O–H)_ band at 1640 cm^–1^. However, the latest band significantly shifts toward low wavenumbers with increasing strength of interaction of water molecules with the surface.^51^ In addition, silica overtones are known to be located at 1640, 1870, and 1960 cm^–1^.^52^ Monodentate bound complexes of fatty acids were identified through vibrational analysis (Δν = ν_C=O_ – ν_C–O_) for several nanosized oxides.^15^ For silica, Δν is 285 cm ^–1^ and corresponds to monodentate complexes.^53^ Based on this and to clarify the mechanisms of interaction of fatty acids with the silica surface, the thermal evolution of the absorption bands in the range of 1400–1900 cm^–1^ for pentanoic acid adsorbed from the gas phase was subsequently investigated. The sample heating temperature range was chosen based on our previous studies. Thermal transformations of pentanoic acid on the surface of silica end at ∼450 °C (Figure 5) as TPD curves of molecular and fragment ions of propylketene show that the desorption of propylketene drops to zero at ∼450 °C.^15,19^ At this temperature, the absorption disappears in the region corresponding to ν_C–O_ and deformation vibrations δ_C–H_ of CH_2_ and CH_3_ groups. This result is consistent with the TPD MS data and confirms the absence of any pentanoic acid on the silica surface at temperatures above 450 °C.

The formation of a much smaller number of dimers and associates on the surface is observed compared to adsorption from the aqueous phase during adsorption from the gas phase (Figure 5). This is evidenced by the decrease in absorption intensity at ∼1720 cm^–1^, which is observed in the spectra in Figure 5 as compared to those in Figure 4. The intensity of the absorption band at 1680 cm^–1^ became almost the same as the intensity of the absorption band of water δ_H–O–H_, since the intensity of the latter decreased due to vacuuming and the fact that adsorption was carried out by the gas-phase method and not from an aqueous solution. However, applying the gas phase adsorption technique did not allow for achieving a clear separation of absorption in the region of ∼1720–1610 cm^–1^ into individual components.

The IR absorption corresponding to ν_C=O_ is present as a shoulder on the water absorption band. Regardless, studying the thermal evolution of these bands from room temperature to 400°C revealed a divergence of two peaks at ∼1615 and ∼1640 cm^–1^, Figure 5. The shift related to the water δ_H–O–H_ bending vibration is related to an increase in the strength of the interaction of the water molecule with the surface.^51^ Furthermore, Schnetzer showed that the lower wavenumber of δ_(H–O–H)_ is observed for water molecules in a highly polarized state.^51^

**Figure 6:**
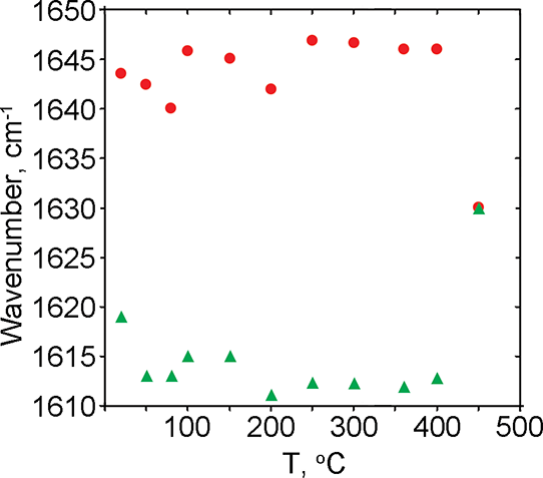
Thermal evolution of the absorption band of the bending vibration of water δ_(H–O–H)_.

The strongest interaction of water molecules with the surface of silica is observed at ∼200 °C since; at this temperature, the lowest value λ_max_ of the absorption band of the bending vibration of water δ_H–O–H_ was recorded, Figure 6. Upon further heating to 400 °C, no additional shift to lower wavenumbers was observed. No splitting of the water band is observed at 450 °C. This implies that all forms of water, including any associated components, have disappeared upon decomposition at this temperature.

**Figure 7:**
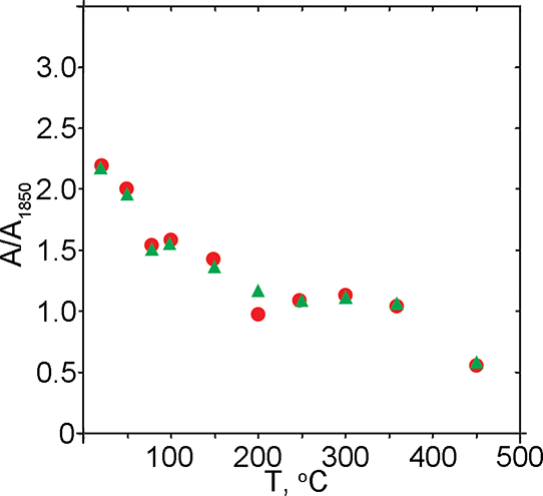
Thermal evolution of the ratio between the intensities of the bending vibration of the water δ_(H–O–H)_ band and the silica overtone band (∼1840–1850 cm^–1^). Red – *A*_1640/1850_ (weakly bound water); green – *A*_1615/1850_ (strongly bound water).

The investigation of pentanoic acid on silica revealed the presence of two distinct forms of water: strongly bound water, which is associated with adsorption complexes, and weakly bound water. Notably, the behavior of water desorption during the temperature-programmed desorption (TPD) process is different, with a significant decrease in the intensity of water desorption observed specifically at the temperature of ketene desorption (∼360 °C). The bands at ∼1636 (δ_H–O–H_) and ∼1860 cm^–1^ (silica overtone) are often used to provide a semiquantitative elucidation of adsorbed water content in the surface layer.^54^ However, they overlap, making an accurate measurement of their areas challenging, Figure 7. From the similar intensities of these bands, one can suggest approximately the same amount of each form. The intensity ratio of these two bands decreased with increasing temperature until 200 °C, Figure 7. At this temperature, it reaches a plateau, and at 450 °C, it gets a value of ∼0.6, which is consistent with literature data for silica materials. Costa et al.^54^ showed that the ratio between the areas of the 1636 and 1860 cm^–1^ bands does not reach zero because the absorbance area at the 1636 cm^–1^ band includes an overtone of silica at ∼1640 cm^–1^.

### 3.3 Quantum Chemical Calculations

**Figure 8:**
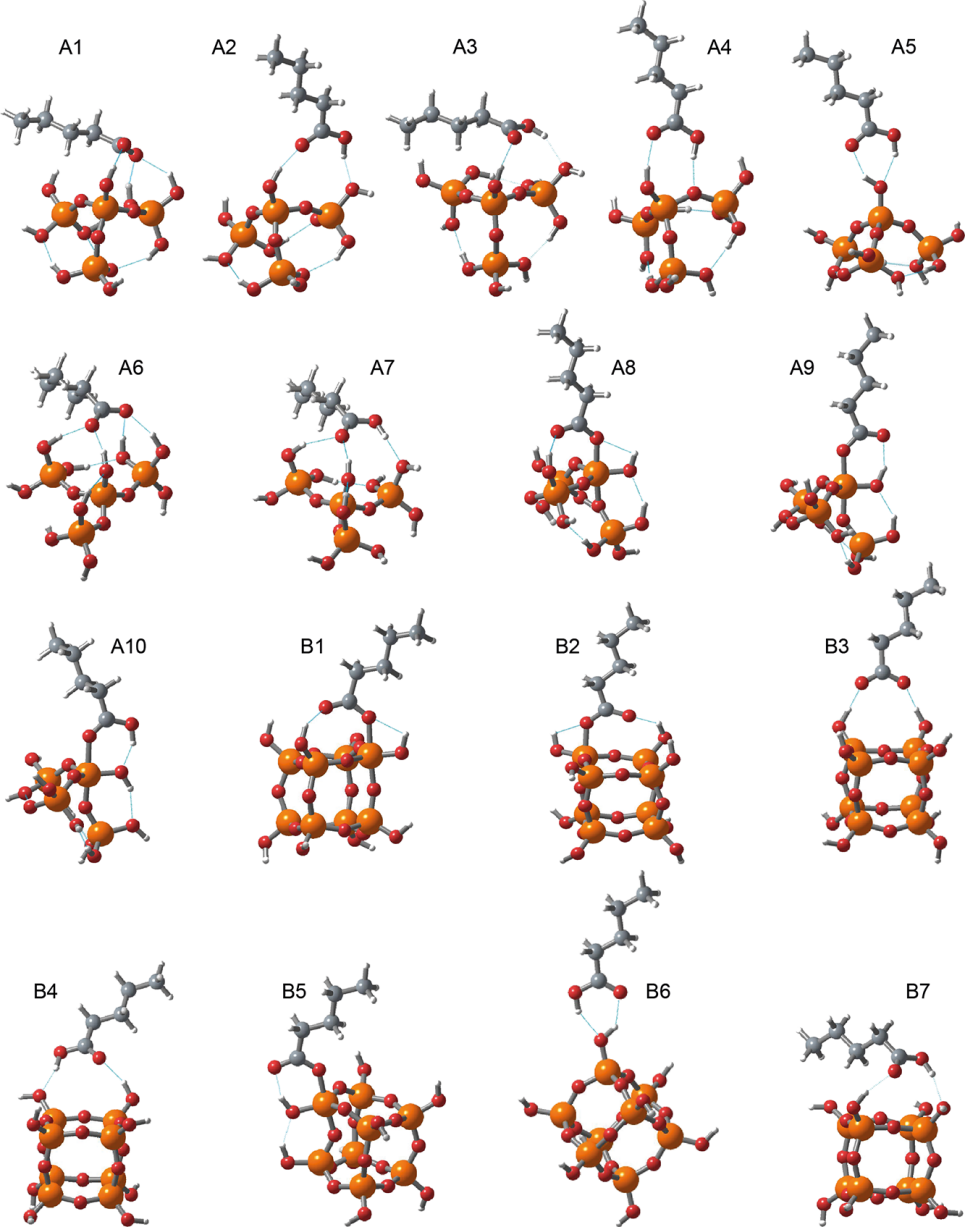
Structural types of surface complexes obtained from DFT calculations.

Table 3 shows the wavenumbers related to the normal vibrational modes of adsorbed complexes on silica. During analysis of the simulated results, special attention was dedicated to identifying the carboxyl group vibrations of valeric acid and its anion (Figure 8). We measured the shift of wavenumbers characteristic of the different surface complexes and associated them with the structural rigidity of the selected models and interacting atoms.

**Table 3:** Values of Total Energy and Normal Vibrations Calculated by the DFT Method for Complexes of the Silica Surface with a Neutral Molecule and an Anion of Pentanoic Acid, which Are Different in Nature, See Figures 8 and 9^a^

structure	acid molecule state	functional	total energy (*E*_tot_), au	frequency, cm^–1^	IR intensity, (km/mol)
A1*	anion	B3LYP	–2489.834440	1620	853.49
		wB97XD	–2489.328791	1683	543.45
		M062X	–2489.236709	1697	560.08
A2	neutral	B3LYP	–2490.347200	1775	414.35
		wB97XD	–2489.831432	1815	449.48
		M062X	–2489.735112	1817	367.45
A3	neutral	B3LYP	–2490.347263	1769	404.37
		wB97XD	–2489.829392	1811	328.21
		M062X	–2489.737514	1823	335.74
A4	neutral	B3LYP	–2490.341615	1768	311.33
		wB97XD	–2489.830188	1815	327.57
		M062X	–2489.728803	1828	324.86
A5	neutral	B3LYP	–2490.345190	1756	316.68
		wB97XD	–2489.827023	1800	336.59
		M062X	–2489.727780	1817	343.18
A6	anion	B3LYP	–2489.847899	1595	653.78
		wB97XD	–2489.332636	1656	678.70
		M062X	–2489.236515	1660	654.12
A7	neutral	B3LYP	–2490.338512	1725	567.37
		wB97XD	–2489.825515	1771	569.46
		M062X	–2489.728725	1780	583.77
A8	anion (pentacoordinate Si)	B3LYP	–2489.824059	1688	402.06
		wB97XD	–2489.312576	1743	392.33
		M062X	–2489.227235	1753	376.40
A9	anion (pentacoordinate Si)	B3LYP	–2489.828675	1653	364.84
		wB97XD	–2489.314982	1707	394.75
		M062X	–2489.229216	1717	391.56
A10	neutral (pentacoordinate Si)	B3LYP	–2490.327281	1725	606.97
		wB97XD	–2489.817746	1763	617.02
		M062X	–2489.726480	1768	669.66
B1**	anion (pentacoordinate Si)	B3LYP	–4174.238445	1661	522.59
		wB97XD	–4173.475992	1716	523.87
		M062X	–4173.407924	1684	591.30
B2	anion (pentacoordinate Si)	B3LYP	–4174.239087	1669	424.45
		wB97XD	–4173.476429	1718	433.03
		M062X	–4173.408024	1690	500.60
B3	anion	B3LYP	–4174.255982	1615	910.04
		wB97XD	–4173.484618	1671	866.49
		M062X	–4173.405767	1650	1102.22
B4	neutral	B3LYP	–4174.760011	1769	494.35
		wB97XD	–4173.993678	1810	443.49
		M062X	–4173.908980	1828	564.18
B5	anion (pentacoordinate Si)	B3LYP	–4174.247630	1652	428.52
		wB97XD	–4173.484559	1709	462.03
		M062X	–4173.413773	1721	474.98
B6	neutral	B3LYP	–4174.756579	1757	317.00
		wB97XD	–4173.987810	1803	334.50
		M062X	–4173.904618	1817	340.18
B7	neutral	B3LYP	–4174.759136	1769	469.31
		wB97XD	–4173.996225	1795	377.56
		M062X	–4173.910895	1813	401.69
C1***	anion	M062X	–6756.344541	1632	645.66
C2	anion (pentacoordinate Si)	M062X	–6756.355978	1718	469.13

a Only the values of the frequencies of highly characteristic oscillations where the carboxyl group of pentanoic acid or its anion participates are given. Frequencies were scaled by 1.0. *, Structures of the A series refer to the “soft” surface model. **, B series structures refer to the “hard” surface model. ***, C series structures refer to the extended “hard” surface model (Figure 9).

The band at 1680 cm^–1^ was assigned to the vibrations of the dissociated carboxyl group, as the simulated IR bands agree with the experiments. The band corresponds to the bond between the carboxyl oxygen and the surface silicon atom, which becomes pentacoordinate (Figure 9). As the data in Table 3 show, this obtained result does not depend on the “soft” or “hard” model of the silica surface. However, it is determined only by the reactivity of the pentanoic acid molecule itself and the coordination capabilities of the silicon atoms on the surface. The formation of such a surface complex was also shown by Abdallah and coauthors.^55^ Additionally, the existence of two types of water in the IR spectra until the complete conversion of acid into ketene provides additional evidence of the bonding of acids with strained siloxane bridges.^44^

**Figure 9:**
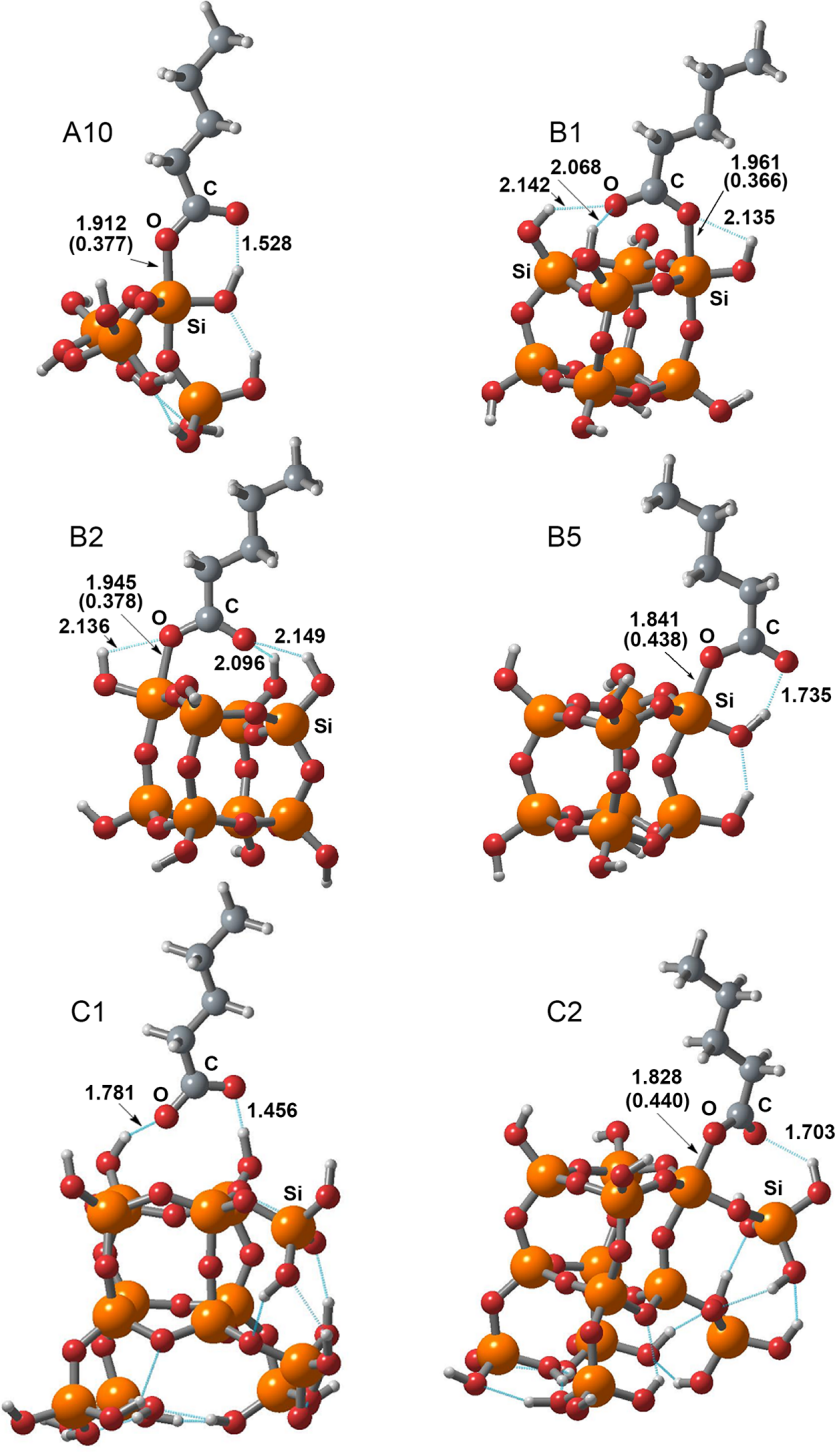
Molecular models for carboxylic acid species adsorbed onto the silica model via the pentacoordinate silicon surface atom.

Figure 8 schematically shows all types of adsorption complexes obtained as a result of DFT modeling. Table 3 shows the values of the total energies (*E*_tot_) of all these complexes calculated by the DFT method as well as the values of the highly characteristic frequencies of the carboxyl group of pentanoic acid or its anion and the intensity of these frequencies.

Analysis of *E*_tot_ values (Table 3) for all structures shown in Figure 8 allowed us to conclude that for structures of the A1–A10 group; the energetically most favorable structures are those in which pentanoic acid or its anion binds to the silica cluster through hydroxyl groups. The situation is different for the “hard” surface model, structures B1–B7. The difference is that in the case of an anion of an acid, this form of adsorption becomes energetically more favorable (Figure 8, structure B5), when the carboxyl group directly interacts with one of the silicon atoms of the surface. With this type of interaction, silicon becomes penta-coordinated. For example, for the M062X functional, the B5 structure is 5.02 kcal·mol^–1^ (preferable to B3 where intermolecular bonding occurs due to hydrogen bonds).

Figure 9 shows a summary of basic geometric parameters for all surface complexes where penta-coordinated silicon is formed. The magnitudes of the orders of coordination bonds between the oxygen of the carboxyl group and the corresponding silicon on the silica surface are also indicated. Such values of bond orders unambiguously confirmed the formation of penta-coordinated silicon.

Structure C1 is a surface complex formed by hydrogen bonds. The bond lengths (functional M062X) are indicated in Angstroms, and in parentheses are the bond orders obtained from the theory of natural bond orbital analysis, using NBO version 3.^56^

This highly relevant result confirmed the hypothesis of the formation of penta-coordinated silicon, with additional DFT studies carried out for the extended model of the “hard” surface. Based on the extended model of the “hard” surface, the results on the formation of pentacoordinate silicon as a result of the adsorption of pentanoic acid anion on the structures of group B were fully confirmed. In addition, the lengths of the coordination bond between the oxygen of the carboxyl group and the surface silicon became significantly smaller (Figure 9) and the values of the bond orders became larger. These results further support the fact that the extension of the surface model led to even more favorable complexes with penta-coordinated silicon. Here it is appropriate to emphasize once again that only structures with penta-coordinated silicon can explain the presence of a band in the region of 1680 cm^–1^ in the experimental IR spectra of pentanoic acid adsorption.

### 3.4 Temperature-Programmed Desorption-Mass Spectrometry (TPD-MS) of Carboxylic Acids

**Figure 10:**
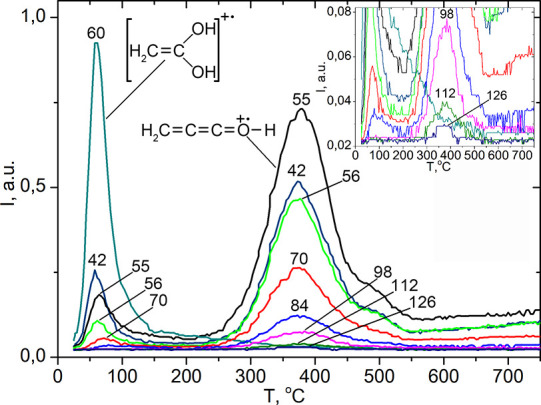
Pyrolysis of silica-supported octadecanoic (stearic) acid (0.3 mmol·g^–1^). TPD curves of fragment ions of the molecular ion of octadecanoic acid with *m*/*z* 60 (the most intense ion in the MS spectra of aliphatic acids), 42, 55, 56, 70, and 84 attributed to the desorption of the physically adsorbed acid. TPD curves of the fragment ions of the hexadecylketene molecular ion with *m*/*z* 55 (the most intense ion in the MS spectra of ketenes) and 42, 56, 70, 84, 98, 112, and 126 obtained during decomposition of the chemically bonded stearic acid fragment.

The high-temperature peak III was observed on TPD-MS curves for ions of hexadecyl ketene with *m*/*z* 42, 55, 56, 70, 84, 98, 112, and 126 (Figure 10). These peaks describe the formation of ketenes upon desorption of octadecanoic acid fragments. It is known that the most intensive ion in the electron impact ionization (electron bombardment ionization, formerly known as electron impact) mass spectra of ketenes is the ion with *m*/*z* 55 [H_2_C=C=C=O–H]^+^.^57^ Peaks related to the destruction of chemisorbed fragment of acids with formation of ketenes [CH_3_(CH_2_)_*n*_–(H)C=C=O]^+^ (for ethanoic acid CH_2_=C=O) where *n* = 0–3; 15 occur on the TPD curve of all saturated aliphatic carboxylic acids. The intensity of ions for this homologous series decreases with increasing molecular mass. An increase in hydrophobicity led to the formation of more surface complexes, and more ketenes developed. This result indicates that the pyrolysis pattern of low molecular weight aliphatic carboxylic acids on the silica surface is also typical for heavier acids. Therefore, this reaction seems to be universal and can be used to process plant raw materials, producing oleochemicals and bulk chemicals for organic synthesis.

Furthermore, data from earlier studies demonstrated that during pyrolysis of carboxylic acids on silica within a homologous series, a direct correlation is present between the maximum acid decomposition temperature with ketene release, and both length and branching of the acid chain.^19,43,58^ Specifically, a longer and more branched acid chain corresponds to a higher decomposition temperature. Interestingly, if such a trend is not exclusive to carboxylic acids, this could imply a possibility to extend such patterns to biomass/waste pyrolysis, particularly those with significant triglyceride fatty acid content. However, it is pertinent to note that Libby et al.'s study did not determine a relationship between chain length and activation energy required for ketene evolution.^21^

The ratio between peak intensities increases in homologous series, although uncorrelated with the substate’s acidity, since the p*K*_a_ remains practically constant over the entire range of compounds studied (Table 3). A shift of the ν_C=O_ absorbance band of more than 60 cm^–1^ to the low-frequency region takes place as compared to that of the monomer (ν_C=O_ = 1757 cm^–1^), and a low perturbation degree of free surface silanols (surface coverage θ ≈ 0.2) testifies favorably to the formation of the surface-adsorbed complex bonded with a bond stronger than hydrogen. Such additional evidence confirms that the acid adsorption on silica occurs via forming a donor–acceptor complex, i.e., n-electrons of the carbonyl’s oxygen bonds with empty Si d-orbitals, which is more favorable with increasing the aliphatic chain length^62^ because this increasing chain length results in increasing electron density on the oxygen atom of the C=O bond. Considering the increase in hydrophobicity, it is observed that the intensity of the ketene peak, observed based on octadecanoic acid, also increases. This correlation suggests that the number of complexes formed on the surface increases with an increase in hydrophobicity. Therefore, the intensity of the ketene peak can serve as an indicator of the enhanced formation of complexes, which is directly related to the hydrophobicity of the system. Probably, it could be associated with complexes formation with the parallel orientation to the surface like on Scheme 1, surface complexes SC IIIa and SC IIIb, because, in this case, the hydrophobicity has a significant impact on the complex stability and, as a result, on their preferable formation on the surface.

**Figure 11:**
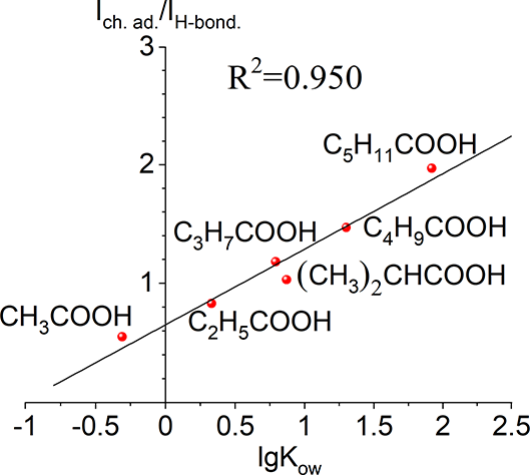
Correlation between the carboxylic acids’ hydrophobic properties (as defined by the *n*-octanol/water partition coefficient lg*K*_ow_) and the intensity ratio *I*_ch. ad._/*I*_H-bond._.

The ratio of TPD-peak intensities (decomposition of adsorpted complex to destruction of hydrogen-bonded complex) is linked with the substrate’s hydrophobicity parameter, lg*K*_ow_, a quantitative measure of the compound’s distribution between aqueous and organic phases from literature data (Table 4). Values of lg*K*_ow_ can be calculated using an additive (atom and group contributions) method, i.e., a molecule is dissected into primary fragments (functional groups or atoms) in which lg*K*_ow_ is known.^60^ Basically, the higher the compound’s hydrophobicity, the more positive value of lg*K*_ow_ is.^60,61^ Calculations of lg*K*_ow_ values for 2-methyl-propanoic and 2,2-dimethylpropanoic acids were carried out using Hansch and Leo’s compilation (additive method).^60^ Indeed, an excellent correlation is observed between the *I*_ch.ad._/*I*_H-bond_ ratio and the hydrophobicity parameter lg*K*_ow_ (Figure 11, Table 4).^60^

#### 3.4.1 Evaluation of Quantity of Each Complex

The percentage of each acid form on the silica surface in Scheme 1 can be calculated by using deconvoluted TPD data. The area under each peak in the TPD curve is proportional to the amount of the corresponding adsorption mode (Table 5). It can be calculated considering the area of each peak as a proportion of the total TPD area as the total amount of acid adsorbed (Table 5).

**Table 4:** Physico-Chemical Parameters of Aliphatic Carboxylic Acids and Ratio of Peak Intensities (Decomposition of Adsorpted Complex to Destruction of Hydrogen-bonded Complex)

acid	chemical formula	*T*_b_, °C	p*K*_a_^59^	lg*K*_ow_	*I*_ch. ad_/*I*_H-bond_
methanoic	HCOOH	100.7	3.75	–0.54	-
ethanoic	CH_3_COOH	117.72	4.756	–0.31	0.55 I(*m*/*z* 42)/I(*m*/*z* 60)
propanoic	CH_3_CH_2_COOH	140.8	4.87	0.33; 0.25	0.83 I(*m*/*z* 56)/I(*m*/*z* 74)
butanoic	CH_3_(CH_2_)_2_COOH	163.25	4.83	0.79	1.18 I(*m*/*z* 55)/I(*m*/*z* 60)
2-methylpropanoic	(CH_3_)_2_CHCOOH		4.84	0.87 calculated according to additive method^60^	1.03 I(*m*/*z* 55)/I(*m*/*z* 60)
pentanoic	CH_3_(CH_2_)_3_COOH	186.35	4.83	1.30 (Et_2_O)	1.47 I(*m*/*z* 55)/I(*m*/*z* 60)
hexanoic	CH_3_(CH_2_)_4_COOH	205.35	4.85	1.92	1.97 I(*m*/*z* 55)/I(*m*/*z* 60)
2.2-dimethylpropanoic	(CH_3_)_3_CCOOH		5.03	1.31 calculated according to additive method^60^	∞
octadecanoic acid(stearic)	C_17_H_35_COOH	376.1	4.5^61^	8.23 (estimated)^61^	∞

**Figure 12:**
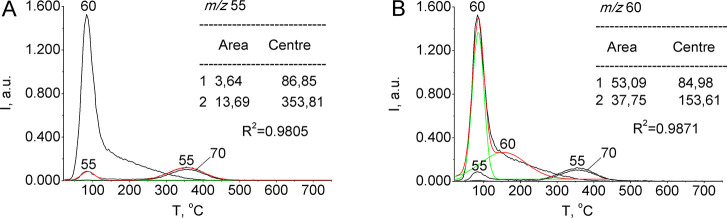
Curve-fitting of deconvoluted TPD-traces of the silica-supported butanoic (butyric) acid sample (0.4 mmol·g^–1^) for ions at *m*/*z* 55 (A) and *m*/*z* 60 (B).

The measurement of areas in the TPD of silica-supported ethanoic acid is illustrated in Figure S3 (Supporting Information). Similarly, the calculations of adsorbed butanoic (Figure 12) and octadecanoic acids (Figure S4 (Supporting Information)) have been conducted for the curve at *m*/*z* 55, i.e., the most intense on ketenes’ thermal desorption curves. There is no peak of hydrogen-bonded acid on TPD-curves of octadecanoic acid, the longest length of the aliphatic chain considered (Figure S4 (Supporting Information)), meaning that the TPD peaks are formed by dimers (complex I) and adsorbed acids.

The semiquantitative data based on TPD areas from the thermolysis of ethanoic, butanoic, and octadecanoic acids are represented in Table 5. It shows that the amount of adsorbed ethanoic and butanoic acids is practically the same, even though the aliphatic chain differs only by two CH_2_ groups. A significant variation is found between ethanoic and octadecanoic acids, which have a chain length difference of 16 CH_2_ groups. In a homologous series of carboxylic acids, the amount of acid chemisorbed on the silica surface increases with increasing hydrocarbon chain length; the total amount of chemisorbed acids is formed by 70% octadecanoic acid, 13% butanoic acid, and 14% ethanoic acid.

**Table 5:** Calculation of the Relative Amount (fraction) of Surface Complexes I (Scheme 1A), II (Scheme 1B), III (Scheme 1C), and IV (Scheme 1D) According to TPD-MS Data^a^

acid, triglyceride, biomass	surface complex	*T*_max_, °C	*m*/*z*	∫(*I*(*m*/*z*))	%	*A*_surf_, mmol·g^–1^	*n*	*E*^#^, kJ · mol^–1^	*k*_0_, s^–1^	Δ*S*^#^, cal·K^–1^ ·mol^–1^	*R*^2^
Fatty Acids											
ethanoic (0.3 mmol·g^–1^)	I	72	60	330	49	0.147	1	72*			
	II	127	60	250	37	0.111	1	83^*^			
	III/IV	326	42	93	14	0.042	1	104	4.27 × 10^6^	–30	0.924
butanoic (0.4 mmol·g^–1^)	I	85	60	53	48	0.192	1	75*			
	II	154	60	38	35	0.14	1	89^*^			
	III/IV	354	55	14	13	0.052	1	104	1.55 × 10^6^	–31	0.930
octadecanoic (0.6 mmol·g^–1^)	I	62	60	38	30	0.18	1	70*			
	II										
	III/IV	380	55	89	70	0.42	1	113	5.04 × 10^6^	–30	0.977
		377	70				1	113	4.49 × 10^6^	–30	0.940
		378	84				1	115	7.21 × 10^6^	–29	0.956
		383	98				1	115	7.20 × 10^6^	–29	0.976
		381	112				1	116	7.07 × 10^6^	–29	0.914
Triglyceride											
glyceryl trimyristate		358	126				1	110	6.16 × 10^6^	–29	0.960
		354	112				1	111	6.99 × 10^6^	–29	0.941
		354	98				1	111	7.77 × 10^6^	–28	0.985
		354	84				1	111	8.73 × 10^6^	–28	0.989
		358	70				1	110	6.60 × 10^6^	–29	0.990
		351	55				1	109	4.32 × 10^6^	–30	0.957
Waste Oil Crop Biomass											
rapeseed meal		∼360	112								
		367	98				1	113	5.30 × 10^6^	–29	0.930
		362	84				1	110	3.24 × 10^6^	–30	0.957
		362	70				1	113	5.72 × 10^6^	–29	0.911
		367	55				1	114	5.14 × 10^6^	–29	0.944

a Kinetic parameters (temperature of maximum reaction rate *T*_max_, reaction order *n*, activation energy *E*^≠^, pre-exponential factor *k*_0_, change of activation entropy Δ*S*^*≠*^) obtained during the pyrolysis of fatty acids, glyceryl trimyristate, and rapeseed meal over silica. *, Calculated approximately from equation *E*^#^= 25*RT*_max_.

### 3.5 Pyrolysis of Triglycerides and Waste Biomass, Rapeseed Meal

In order to confirm that results for fatty acids could be extended for waste biomass (and can be applied to 2G biomass conversion processes), additional studies of the pyrolysis kinetics of trimyristin and rapeseed meal over silica were subsequently conducted.

**Figure 13:**
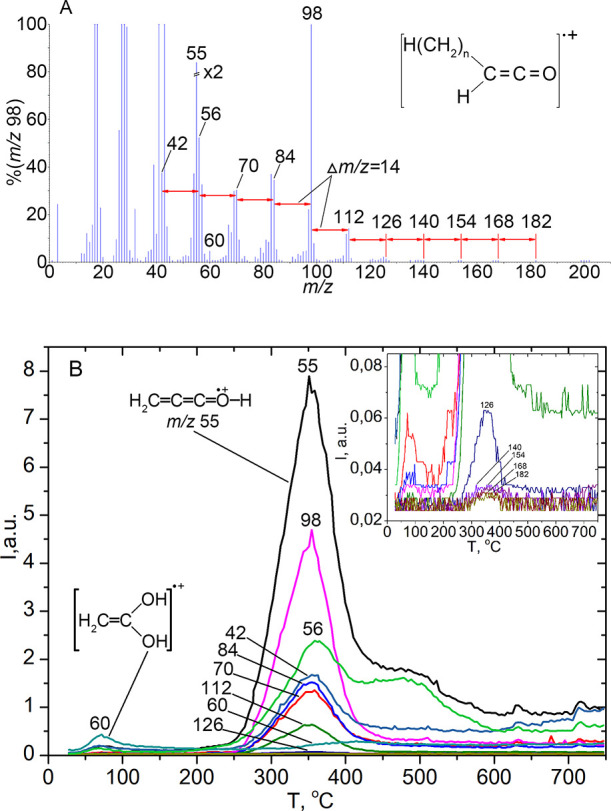
(A) Mass spectra of volatile pyrolysis products at a temperature of 350°C and (B) TPD-curves obtained during pyrolysis of glyceryl trimyristate over silica.

A set of ions characteristic of ketene myristic acid, dodecyl ketene, is observed in the mass spectra of volatile pyrolysis products of glycerol myristic (tetradecanoic) acid over silica at temperatures above 250°C (Figure 13A,B). The most intense ion in the mass spectra of ketenes is [H_2_C=C=C=O–H]^+^ with *m*/*z* 55 and a set of ions with the general formula [H(CH_2_)_*n*_–(H)C=C=O]^+^, which was observed for ketenes synthesized by catalytic pyrolysis from the homologous series of C1···C10 acids.^19,57^ In the case of trimyristin, a set of ions with *n* = 0···10 is observed: *m*/*z* 42, 56, 70, 84, 98, 112, 126, 140, etc., Figure13A,B. At the same time, as the mass of the ion increases, its intensity decreases significantly, especially after *m*/*z* 98, and the molecular ion for long-chain ketenes, as a rule, is not observed, for example, for octyl ketene during pyrolysis of decanoic acid.^19,57,63^ A peak is observed at *T*_max_ ≈ 350°C on the TPD curves for these ions. That is, the temperature of the maximum desorption rate and, accordingly, the activation energy of dodecylketene formation (*T*_max_ = 355°C, *E*^≠^_average_ = 110 kJ·mol^–1^) is lower than that of hexadecylketene (*T*_max_ = 380°C, *E*^≠^_average_ = 114.4 kJ·mol^–1^) in the case of pyrolysis of octadecanoic (stearic) acid (Figure 10, Table 5). This probably indicates that ketene formation occurs due to the transformation of the surface complex of free myristic acid, which is not connected by an ester bond with glycerol (silica is known to effectively hydrolyze ester bonds).

**Scheme 2:**
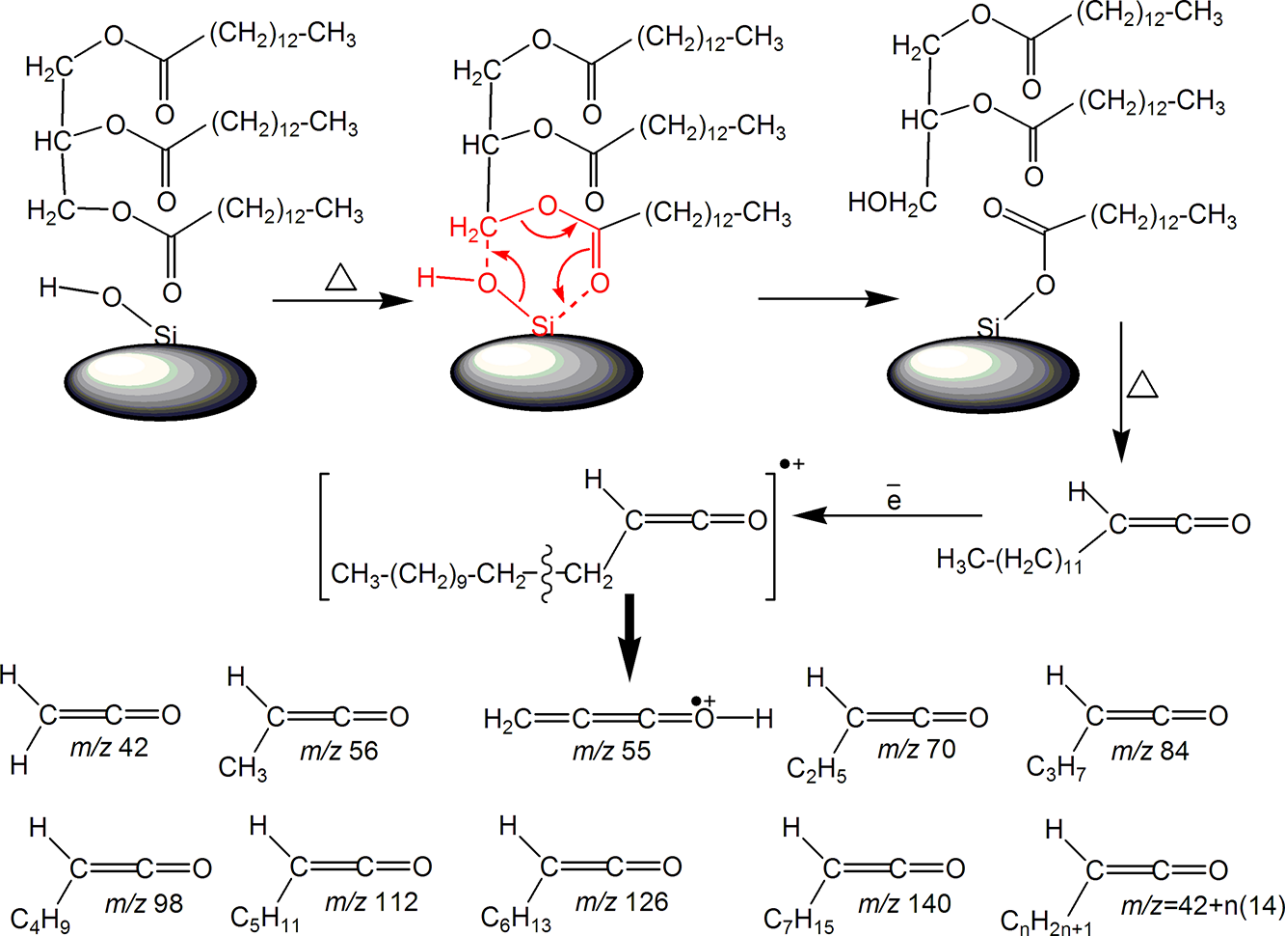
Possible Mechanism of Triglyceride Hydrolysis over Silica with Forming a Surface Complex of Myristic Acid, Which Is Then Subjected to the Ketenization Reaction; EI Fragmentation of Molecular Ion of Dodecylketene and Structures of Its Fragment Ions

The hydrolysis of dicarboxylic esters, including long-chained aliphatic carboxylic acids with high selectivity and yield at temperatures ∼220°C, was carried out by Dyker.^64^ Undoubtedly, trimyristin is also hydrolyzed on the surface of silica when heated to ∼200°C. As we can see (Figure 13B), low intensity peaks on the TPD curves for ions characteristic of free fatty acids, especially for the most intense ion [H_2_C=C(OH)_2_]^+^ with *m*/*z* 60^65^ at a temperature around 100°C, are observed. That indicates the absence of physisorbed and H-bonded forms of the acid (surface complexes I and II, Scheme 1), which were observed for the short-chain and long-chain acids, such as octadecanoic (stearic) (Figures 10 and 12, Table 5). So, hydrolysis mainly occurs during heating, which is absent when triglyceride is loaded on the silica surface. Therefore, triglyceride hydrolysis occurs during heating, forming a surface complex of myristic acid, which is then subjected to ketenization (Scheme 2).

Some publications report on the processes of formation of short-chain fatty acids and some unsaturated aromatic and polyaromatic hydrocarbon due to C–C bond cleavage during fast pyrolysis and thermal cracking of triglycerides.^66^ However, higher pyrolysis temperatures (750 (30 s) and 450 °C (30 s)), higher heating rates of 800°C/min, cracking at high pressures up to 37 MPa, or various catalysts had to be employed.^66^ In our case of pyrolysis on the silica surface, the main process is ketenization. We can monitor if the cracking of triglycerides with C–C bond cleavage and the formation of short-chain acids are evolving by the TPD curve of the most intense characteristic ion with *m*/*z* 60 in the mass spectra of carboxylic acids. From the analysis of the behavior of the TPD curve with *m*/*z* 60, these processes (if taking place at all) occur at higher temperatures (>400°C). Their contribution to the overall pyrolysis process is minimum since the intensity of the ion *m*/*z* 60 contributes with a small percentage as compared to the intensity of the ion with *m*/*z* 55, Figure 13B.

Silica allowed us to avoid the additional stage of hydrolysis of esters in converting 2G biomass rich in triglycerides and lipids. This has a significant economic benefit to develop waste oil crop biomass pyrolysis technologies—especially biomass from rapeseed crops and soybeans, the two largest oil crops grown worldwide.^67^ Rapeseed production reached 29 million tons in 2022. At the same time, the annual production of waste oil crop meal reached 71 million tons.^68^ The rapeseed meal is the primary waste produced via oil pressing. Accordingly, this byproduct has a high triglyceride content. However, this type of biomass is different from lignocellulosic biomass, and therefore, for its effective processing, it requires establishing the patterns of pyrolysis of the main components, particularly fatty acids and triglycerides. The lipid content in rapeseed meals reaches 7–10%, while rapeseed seeds contain a high amount of mono- and polyunsaturated fatty acids (around 90%) and low concentrations (6.5–8%) of saturated fatty acids.^69,70^

**Figure 14:**
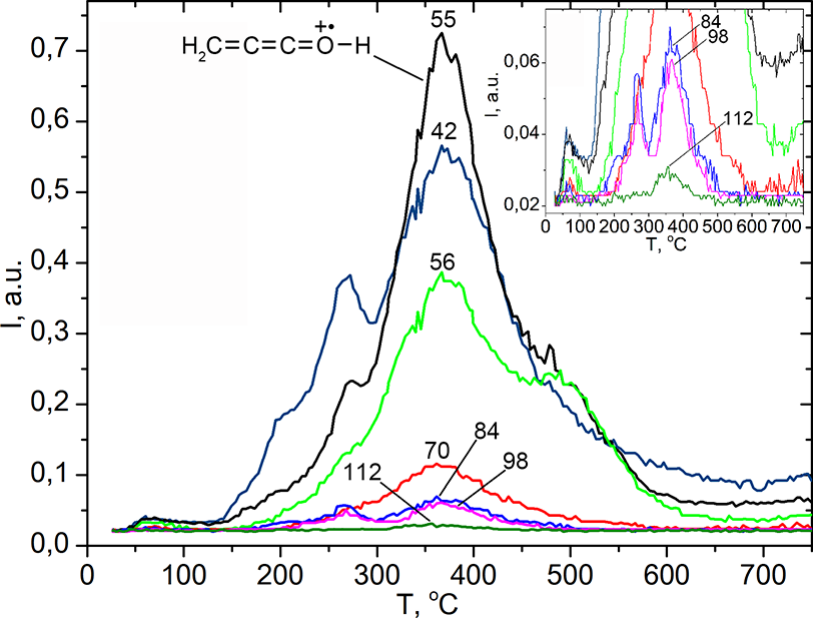
TPD-curves of ketene fragment ions with *m*/*z* 42, 55, 56, 70, 84, 98, and 112 obtained during pyrolysis of rapeseed meal over silica.

**Figure 15:**
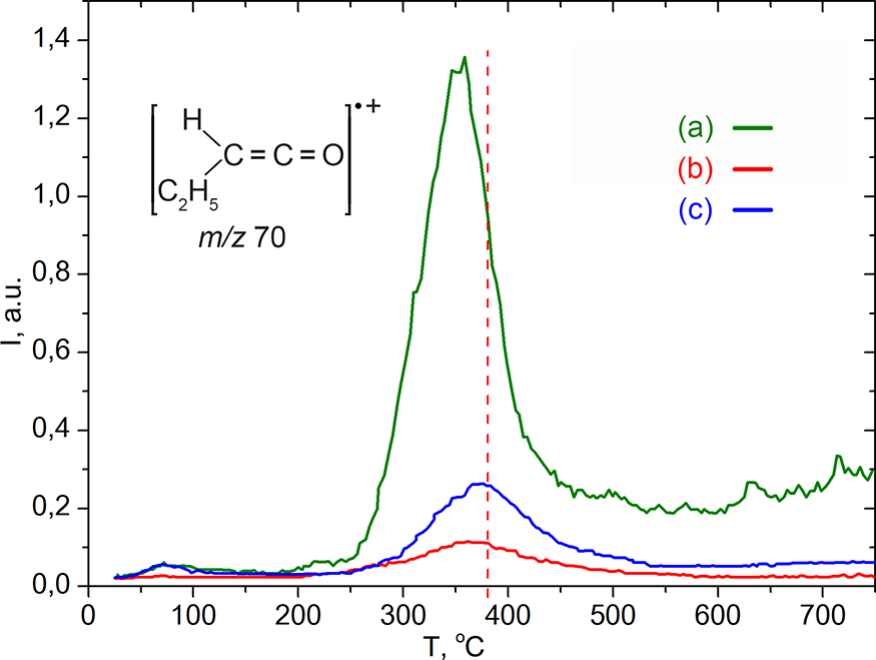
TPD-curves of ketenes fragment ions with *m*/*z* 70 obtained during pyrolysis of (a) glyceryl trimyristate, (b) stearic acid and, and (c) rapeseed meal (*Camelina sativa*) over silica.

The kinetics of pyrolysis of canola meal depend mainly on its fatty acid composition, comprising ca. 60% (*Z*)-octadec-9-enoic acid (oleic acid) (C18:1), 10% (9*Z*,12*Z*,15*Z*)-octadeca-9,12,15-trienoic acid (linolenic acid) (C18:3), 24% (9*Z*,12*Z*)-octadeca-9,12-dienoic acid (linoleic acid) (C18:3), 4% hexadecanoic acid (palmitic acid) (C16:0), 2% octadecanoic acid (stearic acid) (C18:0), 0.25% tetradecanoic acid (myristic acid) (C14:0), and others.^69^ Since almost 95% of the oil consists of acids with a chain length of C18, they will determine the kinetics of canola meal pyrolysis. As can be seen, the temperature of the maximum desorption rate of octadecylketene during pyrolysis of octadecanoic acid (stearic acid) at C18 is about 380 °C, Figure 10, Table 5. That is, this temperature is slightly higher with respect to 370 °C (localization of peaks on TPD curves of the characteristic ions of ketenes with *m*/*z* 55, 70, 84, 98, and 112 during the pyrolysis of rapeseed cake, Figure 14, Table 5). This is quite natural since C16:0 palmitic acid, which in rapeseed oil reaches 6%, also contributes to the formation of these peaks on the TPD curves of ketene fragment ions and, as a result, shifts them to a lower temperature. Our previous work^19^ showed that the temperature of the maximum rate of ketene desorption increases with an increase in the length of the hydrocarbon chain of the acid. Accordingly, the activation energy of the ketenization reaction on the silica surface increases, Figure 15, Table 5. This is precisely why an increase in activation energy is observed in the following order: trimyristat (*E*_average_^≠^ = 110 kJ·mol^–1^) < rapeseed meal (*E*_average_^≠^ = 112.5 kJ·mol^–1^) < stearic acid (*E*_average_^≠^ = 114.4 kJ·mol^–1^), Table 5.

In addition, a “structure–reactivity” correlation was found between *E*^≠^ and Taft induction constants of substituents (*E*^≠^ = 109.6 – 13.457 Σσ*) for the ketenization reaction. On the other hand, in the case of the ketonization reaction over ceria for a homologous series of acids, a correlation between the activation energy and the Taft steric constants of the substituents (*E*^≠^ = 126,84 – 3.096 ΣEs*) was revealed.^14^*T*_max_ is the most sensitive characteristic of the effect of substituents on reaction kinetics. In addition, *T*_max_ reproduced in TPD experiments very accurately; because of this, many approximate equations using *T*_max_ were proposed to calculate approximate values of the activation energy,^19,39,71^ the reaction parameter *p*_o_,^19^ and the kinetic isotope effect.^3,14^

**Scheme 3:**
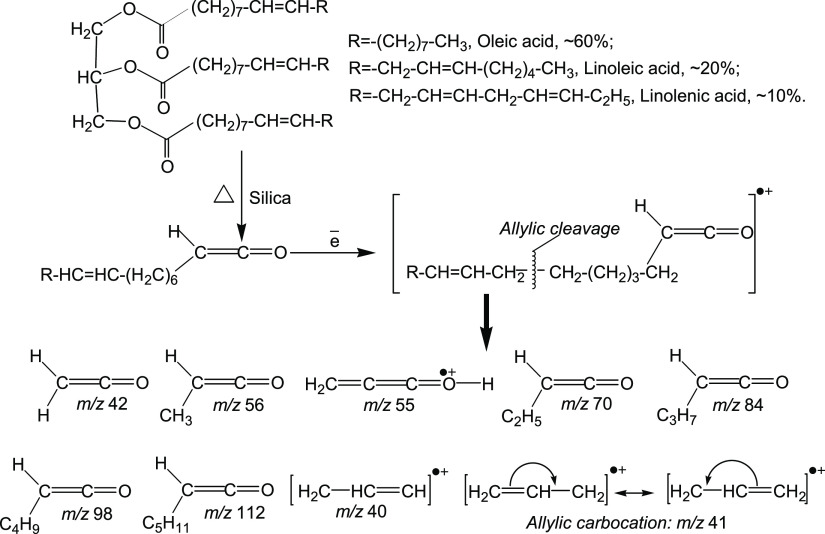
Thermal Decomposition of Triglycerides of Unsaturated Fatty Acids (Oleic, Linoleic, and Linolenic Acids) from Rapeseed Meal; Structure of Fragment Ions of Ketenes Obtained via EI Ionization

In the spectra of volatile products of rapeseed meal pyrolysis over silica, there are no ions larger than *m*/*z* 112 (Figure 14). This is related to the features of fragmentation and ion-molecular reactions of molecular ions of ketenes of unsaturated acids (C18:1, C18:2, and C18:3) under the action of electron impact in the ion source of the mass spectrometer. This feature is due to their structure, particularly double bonds in their molecules, Scheme 3. It is known^72^ that the primary way of alkenes fragmentation under electron impact is C–C bond cleavage in the beta position to the double bond, with the formation of allylic fragments, stabilized due to resonance (Scheme 3). In the case of molecular ions of ketenes of unsaturated acids, the predominant channel of transformation of the molecular ion will be C7–C6 bond cleavage with the formation of fragment ion *m*/*z* 112 (Scheme 3). The patterns obtained for fatty acids, triglycerides, and rapeseed meal in this work are of practical importance and can be used in the future in the development of sustainable technologies for the conversion of 2G biomass with a high content of triglycerides in the processing of oils/fats production waste and related waste feedstocks.

### 3.6 Thermogravimetric Analysis

**Figure 16:**
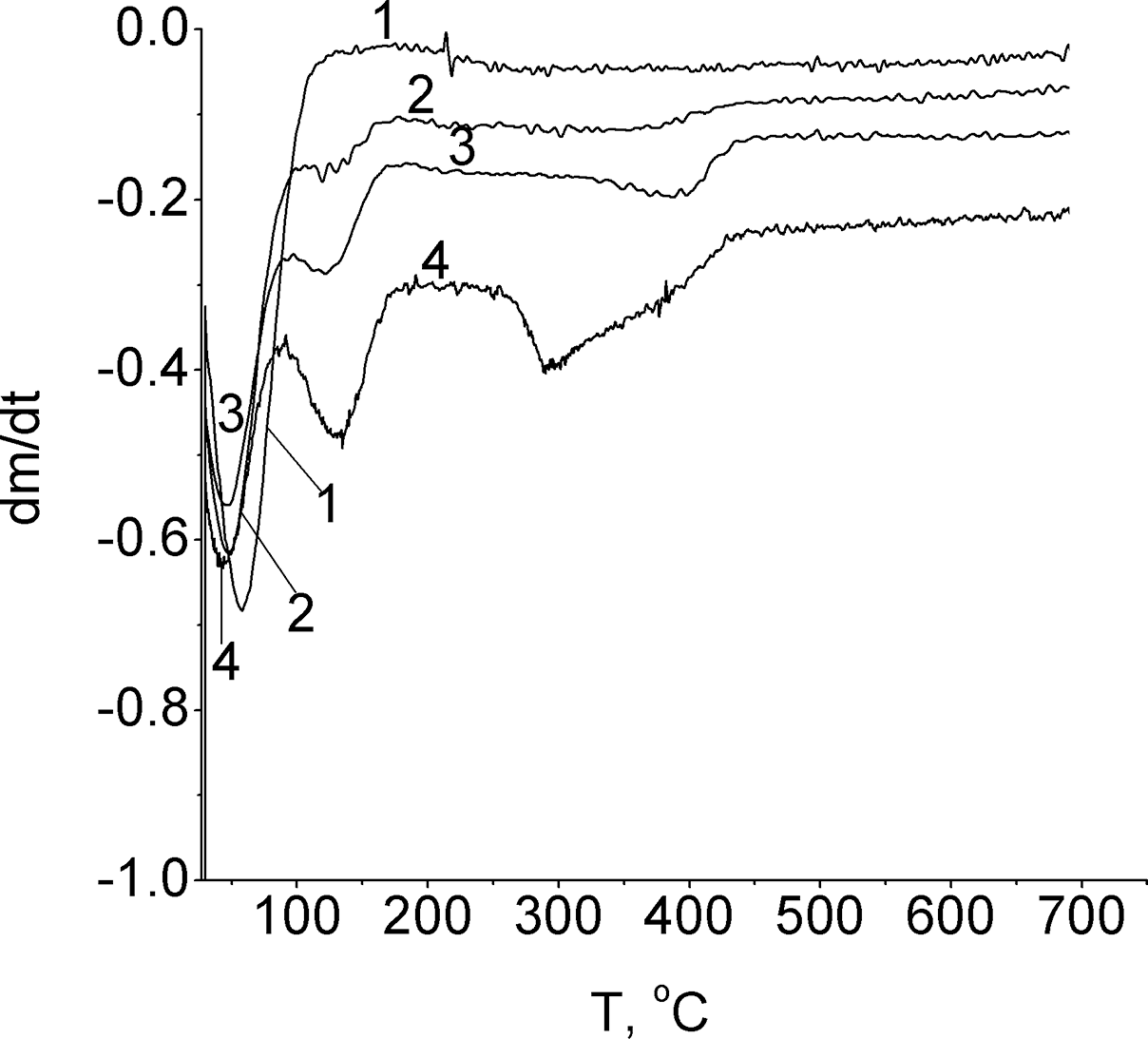
DTG curves of silica (line 1) and silica-supported samples of acids: propanoic (line 2), 2-methylpropanoic acid (line 3), and pentanoic (line 4).

Thermogravimetric analyses of silica and silica-supported acids were performed by soaking them in CCl_4_ solutions. The peaks at *T*_max_ = 62 and 100–200°C and a broader peak at the temperature range of 200–500°C were observed on the derivative thermogravimetry (DTG) curve (Figure 16). The first peak is associated with desorption from the silica surface of the physically adsorbed solvent (CCl_4_) or carboxylic acid dimers. The second one in the temperature region *T*_max_ = 100–200°C would be associated with the decomposition of the hydrogen-bonded acid on the silica surface. The third and fourth peaks are related to the desorption of products from the dehydration and dehydroxylation processes, which take place in the temperate region 200–500°C.

On previous TPD results,^19^ the peak for complex II, *T*_max_, appears in the temperature region 120–150°C (ethanoic – *T*_max_ = 127°C; butanoic – *T*_max_ = 154 °C) and practically coincides with the peak attributed to complex II on DTG curves. In the same way, the decomposition of complexes III/IV on TPD curves in the temperature range 360–390 °C^62^ agrees with the DTG peak (∼390°C) regarding the decomposition of complex IV. Therefore, TPD and DTG indicate a few decomposition stages of adsorbed compounds. In contrast, the differences between these techniques are attributed to the presence of oxygen during DTG thermal decomposition.

## 4 Conclusions

The interaction of linear and branched aliphatic carboxylic acids C1–C6, C18, triglyceride, and rapeseed meal with the fumed silica surface has been studied. Isotherms and physicochemical parameters of short fatty acid C1–C6 adsorption from an aqueous medium were obtained. The formation of different adsorption modes was identified using *in situ* IR spectroscopy, FTIR, thermogravimetry, TPD-MS, and DFT calculations. The observed adsorption modes are dimers bonded with the surface via van der Waals, hydrogen-bonded complexes, and adsorbed complexes bonded on the surface via the C=O group; their relative abundance and decomposition activation energies were obtained. A correlation between the hydrophobicity parameter lg*K*_o/w_ and TPD intensities ratio of peaks assigned to decomposing hydrogen-bonded and C=O bonded complexes for studied acids has been obtained. These results indicate the significant contribution of hydrophobic interactions to the binding of fatty acids with silica. Notably, the formation of surface complexes coordinating the carboxyl oxygen and the silicon atom of the surface leads to a penta-coordinated Si atom, as confirmed by DFT simulations using both “soft” and “hard” model of the silica surface. Moreover, it was strongly confirmed on expanded “hard” model calculations through significantly decreasing the bond length between the Si atom and the O atom of acid. Besides fatty acids, two types of surface water were observed in the IR-spectra (δ_(H–O–H)_ at ∼1640 and ∼1615 cm^–1^), which remained on the surface until all chemisorbed acids were converted into ketenes, indicating the bonding of acids with strained siloxane bridges. Most importantly, these insightful studies support the fact that the established features observed from the ketene formation reaction can also be in principle extended to higher aliphatic acids, triglycerides, and waste biomass. These can pave the way to the design of environmentally safe technologies to process renewable biomass, plant triglycerides, and waste feedstocks into high-value-added products, including fine chemicals and fuels.

## Supporting Information Available

IR spectra of propanoic and hexanoic acids impregnated on the silica surface with different loadings (0.15–0.6 mmol·g^–1^) and the curve-fitted data from deconvoluted TPD-traces for ethanoic and octadecanoic acids supported on silica (PDF)
