# Supplementary material for: The Role of Surface Complexes in Ketene Formation from Fatty Acids via Pyrolysis over Silica: from Platform Molecules to Waste Biomass
Source: J Am Chem Soc. 2023 Dec 4;145(49):26592–610. doi: 10.1021/jacs.3c06966 (PMC10722514; doi:10.1021/jacs.3c06966)
Supplement: Supplementary file 1 — ja3c06966_si_001.pdf [file ja3c06966_si_001.pdf]

Supporting Information

The role of surface complexes in the ketenes formation from fatty acids via pyrolysis over nanosilica

Liana R. Azizova[1,2*], Tetiana V. Kulik[2,3*], Borys B. Palianytsia[2,4], Mykola M. Ilchenko[5], German M. Telbiz[6], Alina M. Balu[4], Sergiy Tarnavskiy[5], Rafael Luque[7,8], Alberto Roldan[3], Mykola T. Kartel[2]

[1]School of Dentistry, Cardiff University; Heath Park, Cardiff, CF14 4XY, UK, AzizovaL@cardiff.ac.uk; Corresponding author.

[2]Chuiko Institute of Surface Chemistry, National Academy of Science of Ukraine, Kyiv 03164, Ukraine.

[3]Cardiff Catalysis Institute, School of Chemistry, Cardiff University, Main Building, Park Place, Cardiff, CF10 3AT, UK.

[4]Departamento de Química Orgánica, Instituto de Química Fina y Nanoquímica, Universidad de Córdoba, Campus de Rabanales, Edificio Marie Curie (C-3), Ctra Nnal IV-A, Km 396, Cordoba, E14014, Spain

[5]Institute of Molecular Biology and Genetics, National Academy of Science of Ukraine, 150 Zabolotnogo Str., Kyiv 03680, Ukraine.

[6]L. V. Pisarzhevsky Institute of Physical Chemistry, National Academy of Science of Ukraine, Nauky Av. 31, Kyiv 03039, Ukraine.

[7]DICEAM, Via Zehender (già via Graziella), Università degli Studi Mediterranea di Reggio Calabria (UNIRC), Loc. Feo di Vito, I89122 Reggio Calabria, Italy.

[8]Universidad ECOTEC, Km. 13.5 Samborondón, Samborondón, EC092302, Ecuador.

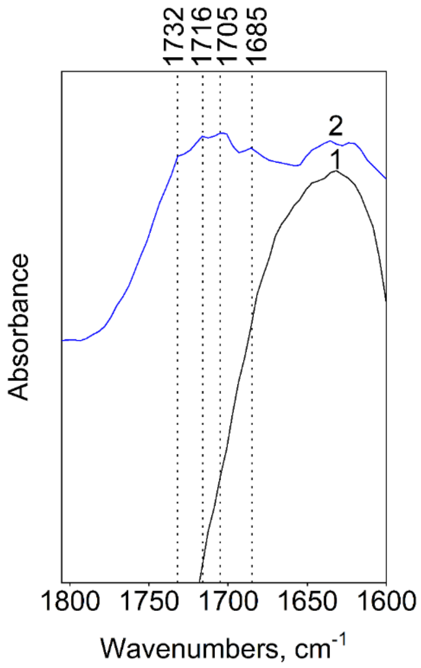

Figure 1S. IR-spectra in the range 1600-1800 cm^{-1} of silica (line 1), silica-supported acids (impregnated) (0.6 $mmol{\cdot}g^{-1}$): hexanoic (line 2).

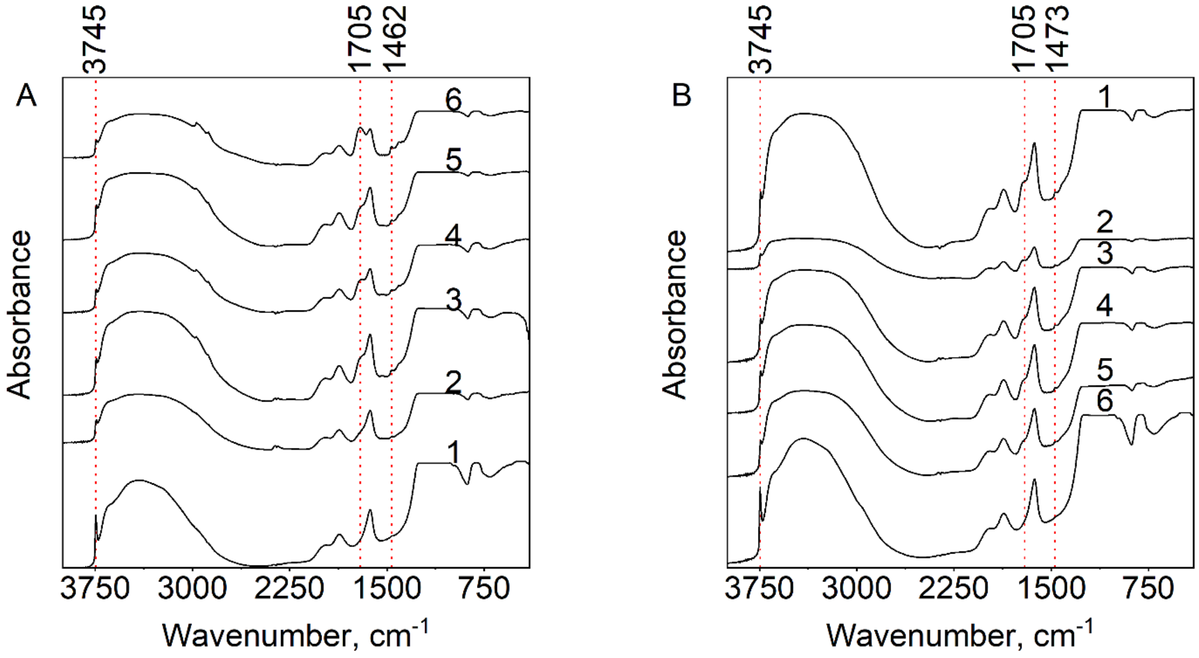

Figure 2S. (A) IR-spectra of silica (line 1) and hexanoic acid impregnated on the silica surface with different loading: 0.15 $mmol{\cdot}g^{-1}$ (line 2); 0.3 $mmol{\cdot}g^{-1}$ (line 3); 0.6 $mmol{\cdot}g^{-1}$ (line 4); 1 mmol·g-1 (line 5); and hexanoic acid adsorbed on the silica surface (0.503 $mmol{\cdot}g^{-1}$; line 6); (B) IR-spectra of silica (line 1) and propanoic acid impregnated on the silica surface with different loading: 0.15 $mmol{\cdot}g^{-1}$ (line 2); 0.3 $mmol{\cdot}g^{-1}$ (line 3); 0.6 $mmol{\cdot}g^{-1}$ (line 4); 1 $mmol{\cdot}g^{-1}$ (line 5); and hexanoic acid adsorbed on the silica surface (0.503 $mmol{\cdot}g^{-1}$; line 6).

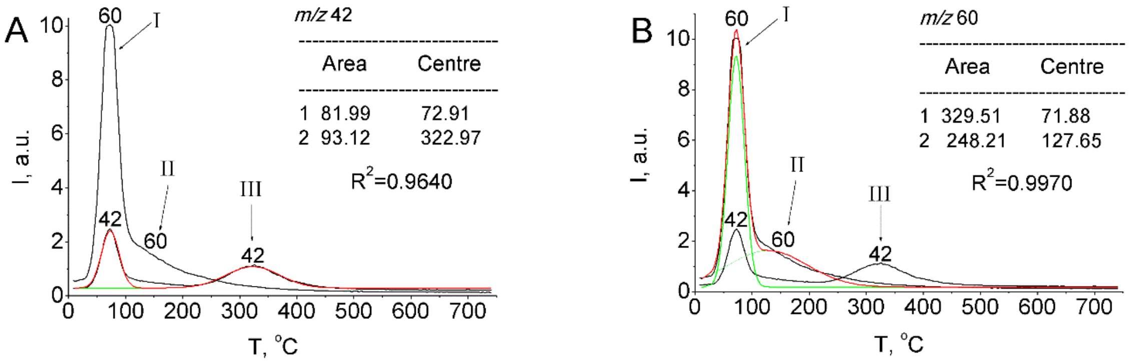

Figure 3S. The curve-fitting of deconvoluted TPD-traces of silica-supported ethanoic (acetic) acid sample (0.3 mmol·g^{-1}) for ions at *m/z* 42 (A) and *m/z* 60 (B).

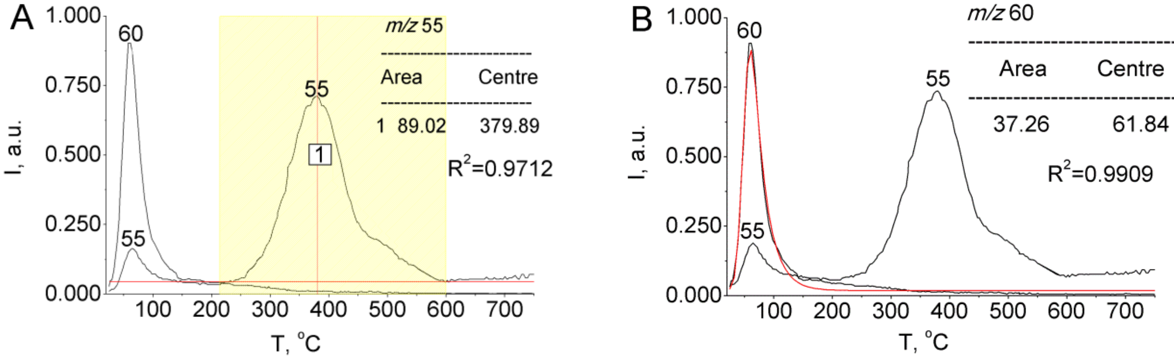

Figure 4S. The curve-fitting of deconvoluted TPD-traces of silica-supported octadecanoic (stearic) acid sample (0.6 mmol·g^{-1}) for ions at *m/z* 55 (A) and *m/z* 60 (B).
